# Influence of Oil and Gas End-Use on Summertime Particulate Matter and Ozone Pollution in the Eastern US

**DOI:** 10.1021/acs.est.4c10032

**Published:** 2024-10-17

**Authors:** Karn Vohra, Eloise A. Marais, Ploy Achakulwisut, Gongda Lu, Jamie M. Kelly, Colin Harkins, Brian McDonald

**Affiliations:** † Department of Geography, University College London, London WC1E 6BT, U.K.; ‡ Stockholm Environment Institute US, Seattle, Washington 98101, United States; § Cooperative Institute for Research in Environmental Sciences, University of Colorado Boulder, Boulder, Colorado 80309, United States; ∥ NOAA Chemical Sciences Laboratory, Boulder, Colorado 80305, United States

**Keywords:** fine particle pollution, oil and gas consumption, atmospheric composition, summertime ozone, eastern United States, chemical transport model, criteria pollutants, isoprene emissions

## Abstract

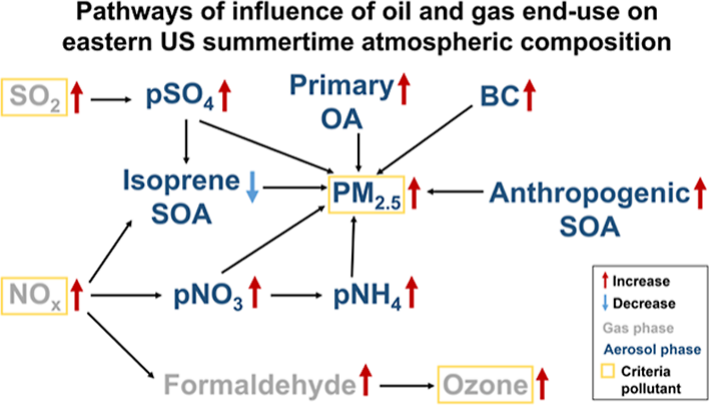

The influence of oil and gas end-use activities on ambient air quality is complex and understudied, particularly in regions where intensive end-use activities and large biogenic emissions of isoprene coincide. In these regions, vehicular emissions of nitrogen oxides (NO_*x*_≡NO + NO_2_) modulate the oxidative fate of isoprene, a biogenic precursor of the harmful air pollutants ozone, formaldehyde, and particulate matter (PM_2.5_). Here, we investigate the direct and indirect influence of the end-use emissions on ambient air quality. To do so, we use the GEOS-Chem model with focus on the eastern United States (US) in summer. Regional mean end-use NO_*x*_ of 1.4 ppb suppresses isoprene secondary organic aerosol (OA) formation by just 0.02 μg m^–3^ and enhances abundance of the carcinogen formaldehyde by 0.3 ppb. Formation of other reactive oxygenated volatile organic compounds is also enhanced, contributing to end-use maximum daily mean 8-h ozone (MDA8 O_3_) of 8 ppb. End-use PM_2.5_ is mostly (67%) anthropogenic OA, followed by 20% secondary inorganic sulfate, nitrate and ammonium and 11% black carbon. These adverse effects on eastern US summertime air quality suggest potential for severe air quality degradation in regions like the tropics with year-round biogenic emissions, growing oil and gas end-use and limited environmental regulation.

## Abstract 2

We elucidate the complex effects of oil and gas end-use activity emissions on air quality in a region where intensive use of oil and gas products and large emissions of biogenic isoprene co-occur.

## 1 Introduction

End use of processed and unprocessed oil and natural gas in fuel combustion and industrial processes contribute significant emissions of air pollutants that are damaging to public health.^1−5^ In the US, oil and gas collectively accounts for more than two-thirds of total energy consumption^6^ and almost all (94%) energy consumed by the transport sector.^7^ High-temperature combustion of fossil fuels in transportation produces large amounts of nitrogen oxides (NO_*x*_)^1,8^ that directly increase abundance of nitrogen dioxide (NO_2_) and that undergo heterogeneous chemistry to form fine particulate matter (PM_2.5_).^9,10^ Other widely used products of oil and gas include industrial and domestic volatile chemical products (VCPs) such as cleaning and personal care products that have been identified as a major source of volatile organic compounds (VOCs) in US cities.^11^ VCPs directly harm health and contribute to PM_2.5_ pollution.^12−14^ Exposure to NO_2_ is linked to increased incidences of childhood asthma^15^ and exposure to PM_2.5_ is linked to premature mortality from multiple causes.^16,17^

Absent is a comprehensive assessment of the primary and secondary effects of oil and gas end-use activities on air pollutant precursor emissions and air quality in environments where end-use activities are in close proximity to large biogenic emissions of isoprene. Previous studies have quantified the adverse effects of end-use of oil and gas together with other fossil fuels such as coal,^18^ so it is not possible to disentangle the contribution from oil and gas end-use. Other studies that have examined air pollution from the oil and gas industry have only assessed the effects of pollution from the production stage of the oil and gas lifecycle.^19−23^ Almost all of these studies have focused exclusively on criteria air pollutant concentrations, rather than investigating the relationship between primary emissions of natural and anthropogenic precursors. This knowledge gap of the complex pathways leading to air quality degradation has implications for regions like the eastern US that are adopting net zero policies and transitioning to cleaner fuels and even more so for fast-growing cities in the tropics with year-round emissions of isoprene that are experiencing rapid industrialization, urbanization, economic and population growth and increasing air pollution.^24,25^

The large influence of urban NO_*x*_ from vehicular combustion on atmospheric composition was apparent during the COVID-19 pandemic, offering a glimpse into urban air quality in the absence of reliance on a prominent oil and gas end-use activity. This event enabled elucidation of the pathways leading to changes in atmospheric composition and improvements in air quality, but in early spring when isoprene emissions are dormant. In the densely populated eastern US, strict lockdowns substantially reduced traffic NO_*x*_ emissions, causing a decline in NO_*x*_ concentrations^26−29^ and affecting abundance of PM_2.5_. Formation of the PM_2.5_ component particulate nitrate (pNO_3_) from oxidation of NO_*x*_ declined.^30−32^ As did the PM_2.5_ component particulate ammonium (pNH_4_). The decrease in pNH_4_ resulted from decline in ammonia emissions from mobile sources and less uptake of ammonia, an acid buffer, to aerosols due to decline in abundance of acidic pNO_3_.^29^ The effect of strict COVID-19 lockdowns on the secondary air pollutant ozone (O_3_) differed in urban and rural areas. Urban O_3_ increased, as its titration by NO_*x*_ was dampened, while suburban and rural O_3_ decreased, as O_3_ production in these environments is limited by the availability of NO_*x*_.^33^

The strict lockdowns only offer an assessment of the contribution of road traffic to O_3_ and PM_2.5_, as VCP usage was unaffected.^29^ The occurrence of these lockdowns in early spring is also only the onset of the spring-summer peak O_3_ pollution period in the eastern US^34^ and precedes the summertime (June-August) peak in biogenic isoprene emissions in the southeast US caused by high temperatures, abundant sunlight, and dense vegetation.^35^ Isoprene is a precursor of multiple air pollutants. These include O_3_, the carcinogen formaldehyde (HCHO), and secondary organic aerosols (SOA) that make a substantial contribution to summertime PM_2.5_.^36−39^ The effect isoprene has on these pollutants depends on the availability of NO_*x*_, as NO_*x*_ modulates the oxidative fate of isoprene.^37,39,40^ In the presence of large concentrations of NO_*x*_ typical of the eastern US, isoprene oxidation leads to high and prompt yields of HCHO and of other similarly small oxygenated VOCs that react to form O_3_.^39,40^ When NO_*x*_ concentrations are relatively low, as may occur in the absence of emissions of vehicular NO_*x*_, formation of SOA precursors via the competing hydroperoxyl radical (HO_2_) oxidation pathway should increase. These precursors include isoprene epoxydiols (IEPOX) that undergo fast acid-catalyzed reactive uptake to aqueous acidic aerosols, glyoxal that oligerimerizes in aqueous aerosols, and low-volatility high-molecular weight products (C_5_-LVOCs) that readily partition to pre-existing aerosols.^36,41,42^ Isoprene SOA formation is most influenced by availability of acidic sulfate aerosols (pSO_4_)^36,43^ that are still abundant and very acidic (pH ∼ 2-3)^44−46^ in the eastern US in summer, despite sustained decline in precursor emissions of sulfur dioxide (SO_2_)^47^ and due to the limited buffering capacity of ammonia.^44^

Here we investigate the complex direct and indirect pathways of influence of emissions of oil and gas end-use activities, hereafter referred to as “end-use”, on summertime eastern US air quality to better inform policies that mitigate emissions from processed and unprocessed oil and gas. To do so, we first process multiple contemporary emissions estimates to obtain an updated inventory of emissions of air pollutant precursors linked to end-use and implement this inventory in the GEOS-Chem chemical transport model to quantify air pollutant concentrations attributable to end-use activities. This includes evaluation of modeled NO_2_, O_3_, PM_2.5_ and PM_2.5_ components with observations from the extensive national surface monitoring network needed to bias correct model-derived attribution of oil and gas end-use activity emissions to air pollutant concentrations.

## 2 Materials and Methods

### 2.1 Oil and Gas End-Use Emissions and the GEOS-Chem Model

Anthropogenic emissions for on- and off-road mobile sources are from the Fuel-based Inventory for Vehicular Emissions (FIVE) (https://csl.noaa.gov/groups/csl7/measurements/2020covid-aqs/emissions/; last accessed 5 September 2022).^8,48^ These are provided as gridded (4-km) hourly emissions for 2018-2020 for use in COVID-19 modeling and emissions studies.^8,29^ Anthropogenic emissions for all other sources, except commercial ships and aircraft, are from the US Environmental Protection Agency (EPA) National Emissions Inventory (NEI). These are provided as county-level annual totals of active Source Classification Codes (SCCs). The 248 SCCs we classify as end-use are listed in Supporting Information Table S1.

The most recent publicly available NEI emissions year not affected by the COVID-19 pandemic is 2017 (NEI 2017; https://www.epa.gov/air-emissions-inventories/2017-national-emissions-inventory-nei-data; last accessed 5 September 2022) and so we select 2017 as the study year. Oil and gas consumption in the US has remained relatively stable since 2017. In 2018-2019, it increased by 5%, whereas in 2020-2021 it declined to 2017 totals due to reduced demand brought on by the COVID-19 pandemic.^7^ We generate gridded hourly emissions from the NEI 2017 emissions using a custom codebase adapted from the Sparse Matrix Operator Kernel Emissions (SMOKE) processing system, previously developed and used in numerous publications.^29,48,49^ SMOKE processing yields hourly emissions for the same grid resolution and days as FIVE. Due to the computational cost of processing NEI, we generate emissions for end-use and other anthropogenic activities for July only. For consistency, we also select the FIVE July hourly emissions. FIVE emissions are for 2018 and so slightly underestimate 2017 emissions, as NO_*x*_ emissions from mobile sources declined steadily each year prior to the pandemic due to controls on emissions.^50^ We use the NEI and FIVE data to calculate emissions for the other summer months and April-May needed for model chemical initialization by applying broad sector-specific seasonal scaling factors derived with the NEI 2016 emissions that have been custom built for use in GEOS-Chem (http://geoschemdata.wustl.edu/ExtData/HEMCO/NEI2016/v2021-06/, last accessed 10 December 2023). We regrid the FIVE and NEI 4-km resolution emissions to 0.1 ° × 0.1 ° to implement in GEOS-Chem.

We use GEOS-Chem version 13.0.0 (10.5281/zenodo.4618180; last accessed 1 March 2023) to simulate the complex changes in atmospheric composition linked to emissions from end-use activities. The model is driven with NASA GEOS-FP meteorology. We simulate the model in a nested configuration over contiguous US (23 °-51 °N, 128 °-63.5 °W) at 0.25 ° × 0.3125 ° (∼28 km latitude × ∼27 km longitude at the nested domain center) in summer (June-August 2017). Dynamic (3-hourly) boundary conditions are from a global simulation (4 ° × 5 °). Model chemical initialization is achieved with a 1-year spinup for the boundary conditions and 2 months for the nested domain.

NEI 2017 emissions of all aerosol-bound metals and "Other PM_2.5_" are emitted as dust. Anthropogenic emissions outside the US, including shipping emissions in US territorial waters, are from the global Community Emissions Data System (CEDS) v2 emission inventory. Aircraft emissions are from the global Aviation Emissions Inventory Code (AEIC) inventory for 2005.^51,52^ The model also includes open fire and natural emissions. Open fire emissions are from the Global Fire Emissions Database (GFED) version 4 with small fires. Natural emissions are calculated using the Model of Emissions of Gases and Aerosols from Nature (MEGAN) version 2.1^35^ for biogenic VOCs, and the parametrizations of Hudman et al.^53^ for soil NO_*x*_, and Jaeglé et al.^54^ for sea salt. Hourly offline dust^55^ and lightning NO_*x*_^56^ emissions are those generated at 0.25 ° × 0.3125 °. The timezone file currently implemented in GEOS-Chem, derived using Voronoi polygons, is at coarser resolution (1 ° × 1 °) than the nested grid and defines timezones by standard time only. We update GEOS-Chem representation of timezones by gridding to 0.1 ° × 0.1 ° the timezone data from the Time Zone Database (https://www.iana.org/time-zones; last accessed 16 April 2023) and by also accounting for daylight savings time, thus shifting emissions from sources like vehicle traffic to 1 hour earlier than standard time.

The model includes detailed coupled gas- and aerosol-phase chemistry and wet and dry deposition to represent formation and loss of O_3_ and PM_2.5_ components. pSO_4_ is formed from oxidation of SO_2_ by the hydroxyl radical and by in-cloud hydrogen peroxide.^10^ Formation of pNO_3_ and pNH_4_ is via thermodynamic equilibrium calculated by ISORROPIA II.^57^ We use a revised parametrization of rainout and washout^58^ that increases wet scavenging of pNO_3_ and pNH_4_ in the US. Primary OA (POA) is emitted as 50% hydrophilic and 50% hydrophobic^59^ that is assumed to age to hydrophilic with a lifetime of 1.15 days.^60,61^ SOA from isoprene is calculated using a mechanism that accounts for irreversible reactive uptake of the isoprene oxidation products IEPOX, C_5_-LVOCs, and glyoxal to aqueous aerosols.^36^ IEPOX, the dominant isoprene SOA precursor, undergoes acid catalyzed reactive uptake, so depends on the abundance of acidic aerosols. SOA from monoterpenes and sesquiterpenes is represented in the model using fixed mass yields of 10% each.^62^ Anthropogenic SOA formation from oxidation of anthropogenic VOCs and from aerosol uptake of intermediate- and semi-volatile organic compounds (IVOCs and SVOCs) is estimated as 6% by mass of anthropogenic CO emissions.^63^

The nested model is simulated with and without end-use emissions. End-use emissions turned off in the latter are FIVE for on-road and off-road mobile sources, NEI 2017 emissions of the selected end-use SCCs (Supporting Information Table S1 ), AEIC for aircraft, and CEDS for shipping. The difference between the two simulations is used to quantify the contribution of end-use to summertime O_3_ and PM_2.5_ in eastern US (east of 100 °W).

### 2.2 Surface Air Pollutant Observations to Evaluate GEOS-Chem

The eastern US includes a widespread, dense network of measurements of surface concentrations of PM_2.5_, PM_2.5_ components (organic carbon (OC), pNO_3_, pNH_4_, and pSO_4_), NO_2_, and O_3_ for extensive model evaluation and to correct model biases where these impart errors in findings that have implications for policy decisions. The network measurement data are from the US EPA Air Quality System (AQS) database (https://aqs.epa.gov/aqsweb/documents/data_api.html; last accessed 1 March 2023) for both trace gases and aerosols and the Interagency Monitoring of Protected Visual Environments (IMPROVE) program (http://vista.cira.colostate.edu/Improve/; last accessed 16 April 2023) for aerosols only. AQS sites, located mostly in urban and suburban areas, are part of the national ambient air monitoring program to assess compliance with the national air quality standards. IMPROVE monitors visibility in areas with special air quality protections such as national parks and wilderness areas. We use air quality data for all sites operational in the eastern US in June-August 2017. PM_2.5_ measurements are from beta attenuation monitoring or gravimetric sampling, OC from thermal optical reflectance analysis, pNO_3_ pNH_4_, and pSO_4_ from ion chromatography, NO_2_ from chemiluminescence, and O_3_ from an ultraviolet detector. We select sites with 75% data coverage in each month totalling 423 for PM_2.5_, 161 for OC, 154 for pNO_3_, 108 for pNH_4_, 159 for pSO_4_, 229 for NO_2_, and 829 for O_3_.

We correct OC observations from the IMPROVE network for a ∼30% low bias attributed to evaporative loss of semivolatiles.^64,65^ NO_2_ observations from chemiluminescence instruments used as part of air quality monitoring networks are often denoted as “NO_2_*” to represent positive interference from decomposition of thermally unstable NO_*x*_ reservoir compounds such as peroxy-acetyl nitrate (PAN), methyl peroxy nitrate (MPN, CH_3_O_2_NO_2_) and peroxy nitric acid (HNO_4_).^66,67^ We calculate the equivalent NO_2_* in GEOS-Chem using reported operating temperature-dependent percentage interference values.^67^ At typical operating conditions of ground-based chemiluminescence instruments, interference includes reservoir compounds such as 5% PAN, 100% MPN and 100% HNO_4_. Interference according to GEOS-Chem is small (<1%), as these thermally unstable reservoir compounds are not prevalent in summer in the eastern US.

For consistent comparison of GEOS-Chem and the ground-based observations, we sample outputs from the lowest model layer and grid the observations to the GEOS-Chem grid. Modeled PM_2.5_ is calculated as the sum of the concentrations of individual simulated components multiplied by hygroscopic growth factors at the same relative humidity (35% RH) and temperature and pressure (ambient) as the measurements^68^
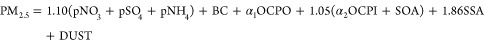
1

Equation 1 terms are BC for black carbon, OCPO for the hydrophobic component of primary OC, OCPI for the hydrophilic component of primary OC, SOA formed from biogenic and anthropogenic precursors, SSA for accumulation-mode sea salt, and DUST is dust aerosol. The α terms are OA/OC ratios that are typically 1.5 for primary sources and 2.1 after aging.^69^ OCPO is freshly emitted and so we use α_1_ = 1.5. OCPI is a mix of freshly emitted and aged aerosol and so we use α_2_ = 1.8. We sample hourly O_3_ concentrations from GEOS-Chem to calculate maximum daily 8-h running-mean ozone (MDA8 O_3_), the metric used for air quality compliance monitoring and health burden assessments.

## 3 Results and Discussion

### 3.1 Contribution of Oil and Gas End-Use Activities to Emissions

**Figure 1:**
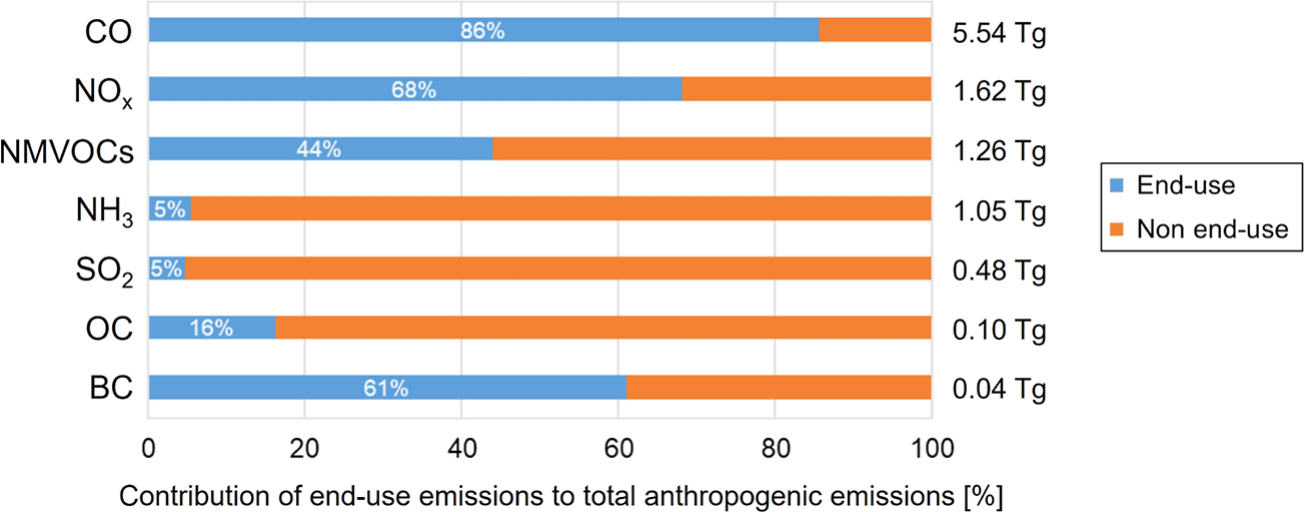
Eastern US anthropogenic air pollutant precursor emissions in summer 2017. Stacked bars show the relative contribution of end-use (blue) and non end-use (orange) emissions to total anthropogenic emissions for dominant gaseous pollutants (CO, NO_*x*_, NMVOCs, NH_3_ and SO_2_) and primary PM_2.5_ components (OC and BC). Values outside the bars are the total emissions for June-August. Value for NMVOCs is total speciated emissions included in GEOS-Chem (Section 2.1).

Figure 1 shows total and end-use contribution to anthropogenic emissions of dominant air pollutant precursors in the eastern US for June-August 2017 that we include in GEOS-Chem. All NEI and FIVE emissions of CO, NO_*x*_, ammonia (NH_3_), SO_2_ and primary PM_2.5_ are included in the model, whereas a portion of NMVOCs emissions are represented in GEOS-Chem. Even though there are only 17 lumped and individual NMVOCs in GEOS-Chem, these account for 65% of the NMVOCs mass reported by the inventories. The largest anthropogenic emissions in GEOS-Chem are for CO (5.5 Tg), NO_*x*_ (1.6 Tg) and NMVOCs (1.3 Tg). End-use activities contribute to more than half the emissions of CO (4.7 Tg), NO_*x*_ (1.1 Tg), and BC (23 Gg), and just below half for NMVOCs (0.6 Tg). Dominant end-use activities include all mobile (vehicle) sources for CO^70,71^ and NO_*x*_,^72^ diesel vehicles for BC,^72,73^ and VCPs for NMVOCs.^11^ The relative contribution of end-use activities to anthropogenic emissions of the other compounds is smaller at 16% for OC (17 Gg), and 5% for NH_3_ (57 Gg) and for SO_2_ (23 Gg). End-use emissions are mainly from catalytic converters of onroad gasoline for NH_3_,^74^ industrial boilers and commercial buildings for SO_2_,^75^ and mobile sources for OC.^76^ There is a very small contribution of end-use activities to inorganic primary PM_2.5_ components (not shown) of 1.7 Gg pSO_4_, 0.7 Gg pNH_4_, and 0.3 Gg pNO_3_.

### 3.2 Addressing GEOS-Chem Biases in Particulate Nitrate and Ammonium

**Figure 2:**
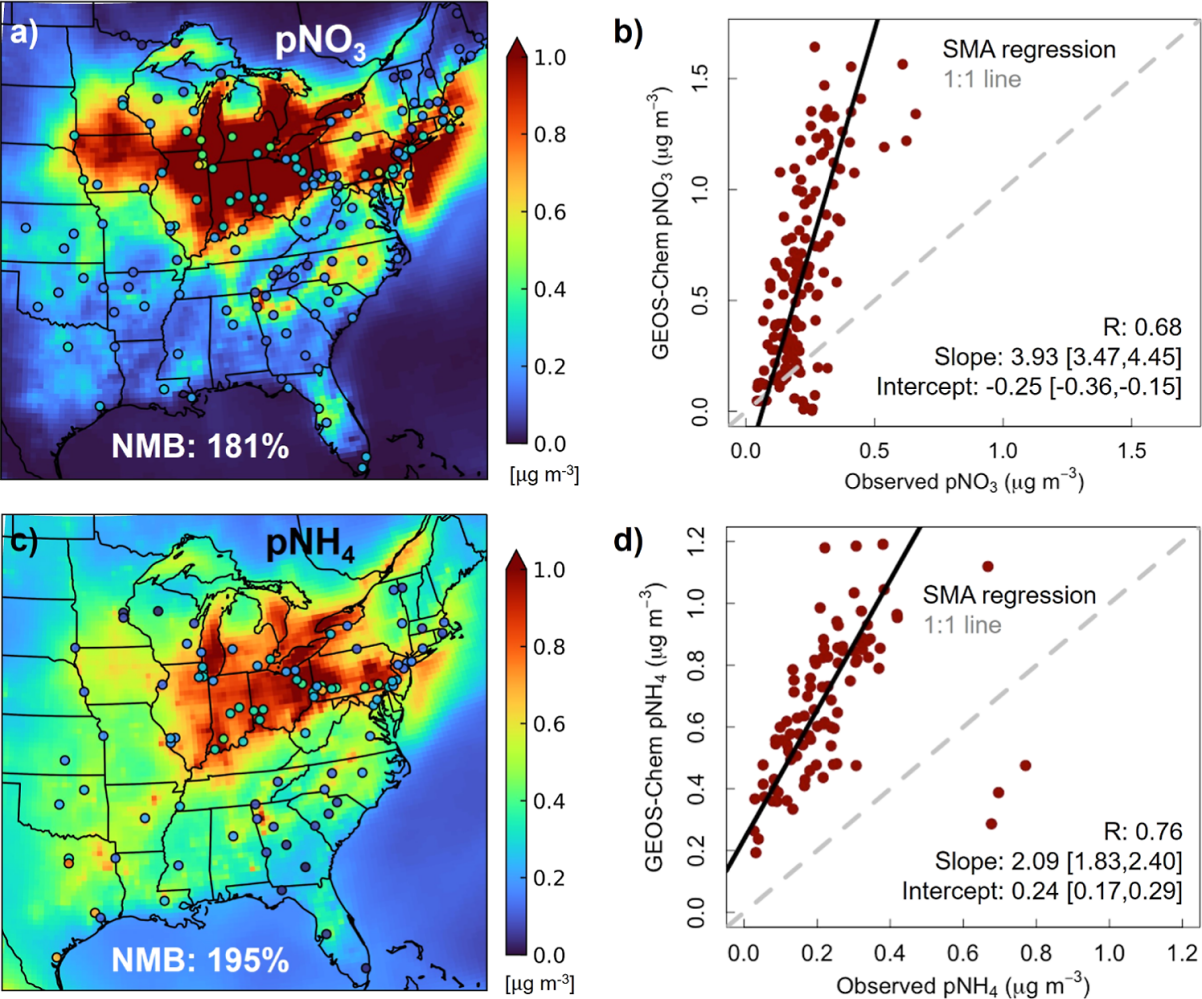
Assessment of GEOS-Chem eastern US summer 2017 surface pNO_3_ and pNH_4_. Maps compare simulated (background) and observed (circles) June-August mean pNO_3_ (a) and pNH_4_ (c). Values inset are the model NMB for coincident grid squares and observations. Scatter plots compare coincident modeled and observed pNO_3_ (b) and pNH_4_ (d). Lines are SMA regression (black solid) and 1:1 agreement (gray dashed). Values inset are Pearson’s correlation coefficients (*R*) and SMA regression statistics. Relative errors on the slopes and intercepts are the 95% CI. *R* and SMA regression in panel (d) exclude the 3 outlier points in Texas.

GEOS-Chem comparison to surface observations is shown and discussed in the SI (Supporting Information Text S1 and Figures S1-S2 ) for pollutants (NO_2_, MDA8 O_3_, PM_2.5_, OC) that the model is consistent with spatially (*R* > 0.5). Pertinent for perturbation studies is that the model also reproduces the observed variance for these pollutants, yielding regression slopes close to unity. The model exhibits a large and significant bias in both pNO_3_ and pNH_4_ (Figure 2). Observed pNO_3_ and pNH_4_ are typically <0.5 μg m^–3^ in the eastern US in summer. GEOS-Chem overestimates each component by almost a factor of 3 (NMBs of 181% for pNO_3_ and 195% for pNH_4_). Such large biases have also been reported in prior studies targeting the contiguous US^86,87^ and other parts of the world like China that have substantial anthropogenic precursor emissions of pNO_3_ and pNH_4_.^88^ A range of potential causes have been suggested for the bias in pNO_3_ that in turn enhances NH_3_ partitioning to aerosols to form pNH_4_, as the additional aerosol acidity from pNO_3_ promotes partitioning of gas-phase NH_3_ to the aerosol to neutralize the acidity by forming pNH_4_. Causes that have been proposed for the model bias in pNO_3_ include uncertainties in processes that affect abundance of nitric acid (HNO_3_), the NO_*x*_ oxidation product and precursor of pNO_3_,^86^ kinetic inhibition of pNO_3_ formation by organically coated aerosols not accounted for in GEOS-Chem,^89^ and low biases in pNO_3_ and pNH_4_ wet deposition.^58,87^ Heald et al.^86^ tested sensitivity to uncertainties in a range of processes that affect model simulation of HNO_3_ to find that a brute force 75% decrease in HNO_3_ is most effective at improving agreement between GEOS-Chem and the same IMPROVE surface observations of pNO_3_ and pNH_4_ used in this study. Silvern et al.^90^ found that invoking kinetic inhibition was only effective at addressing the bias in the southeast US. We already implement the more efficient wet deposition scheme of Luo et al.^58^ that we find has limited effect on pNO_3_ and pNH_4_ in summer.

The much greater variance in modeled pNO_3_ (slope ∼ 3.9) and pNH_4_ (slope ∼ 2.1) will cause a large overestimate in attribution of end-use emissions to these aerosol components. So, we apply a correction to modeled pNO_3_ and pNH_4_ to address this bias. To do so, we divide the difference in pNO_3_ and pNH_4_ concentrations between the simulations with and without end-use emissions by the regression slopes in Figure 2b and d. This yields corrected attributable concentrations that we apply to eq 1 to recompute end-use PM_2.5_. This decreases eastern US mean end-use attributable pNO_3_ by 0.21 μg m^–3^ and pNH_4_ by 0.05 μg m^–3^. Accounting for this correction and an associated decrease in aerosol water at 35% RH (eq 1) leads to a 0.32 μg m^–3^ decrease in end-use PM_2.5_. We use corrected end-use pNO_3_, pNH_4_, and PM_2.5_ to determine the influence of end-use emissions on summertime air pollution in the eastern US in the section that follows.

### 3.3 Contribution of Oil and Gas End-Use Activities to Air Pollution

**Figure 3:**
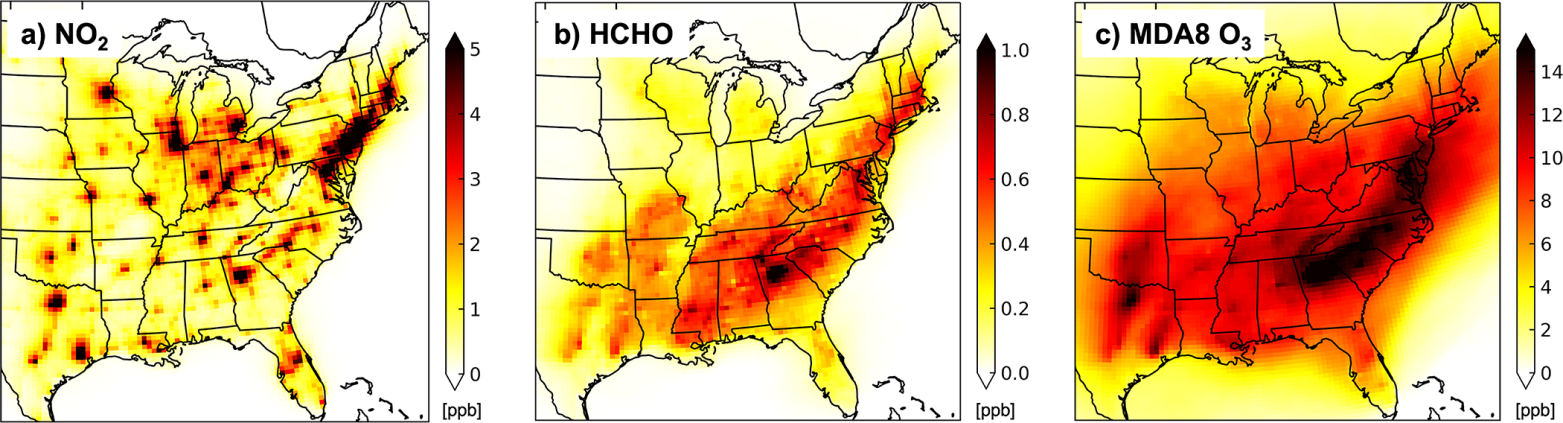
Contribution of oil and gas end-use activities to surface concentrations of ozone and its precursors in summer (June-August) 2017. Maps are of eastern US NO_2_ (a), HCHO (b) and MDA8 O_3_ (c) from the difference in GEOS-Chem simulations with and without end-use emissions.

**Figure 4:**
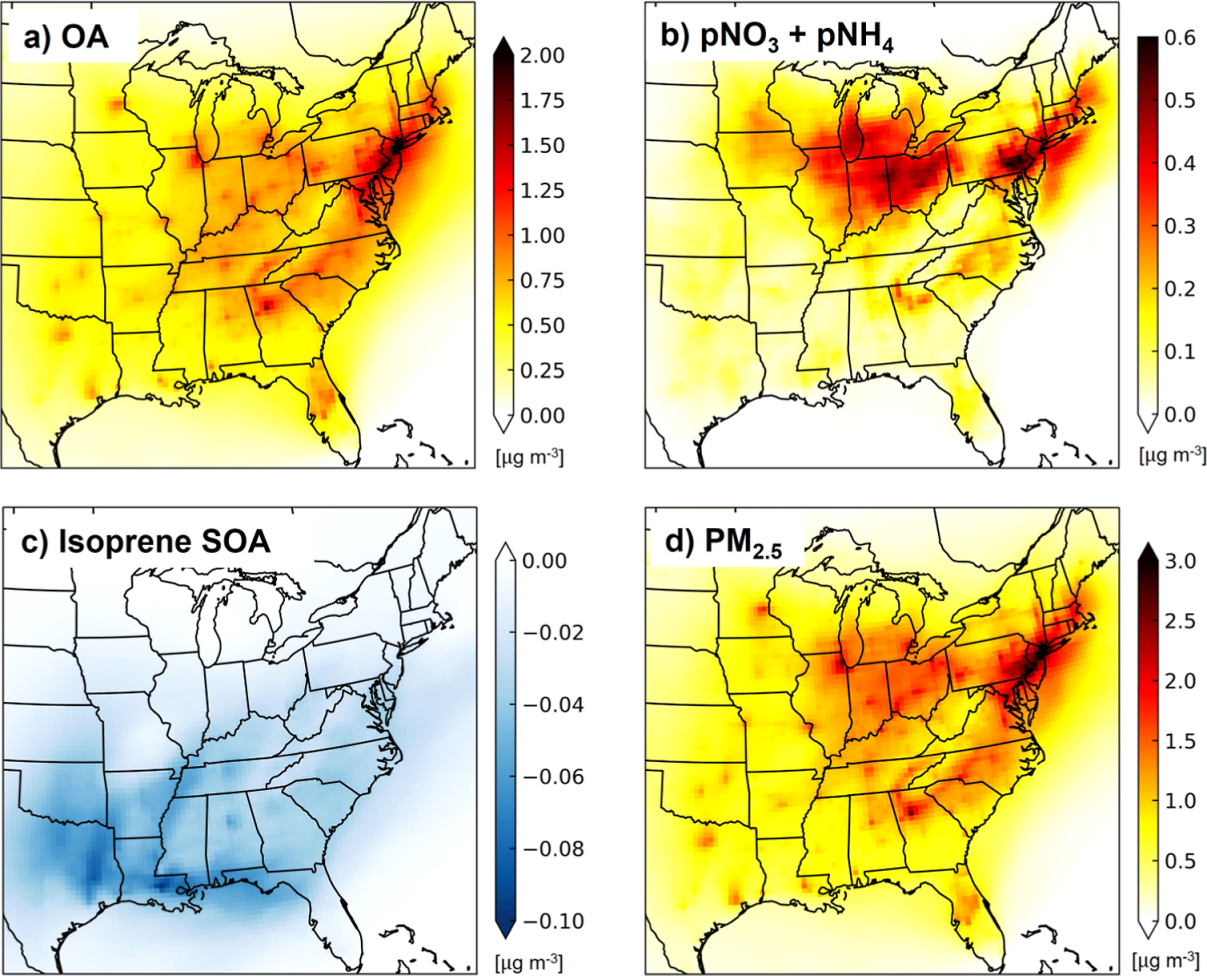
Contribution of oil and gas end-use activities to surface concentrations of PM_2.5_ and its components in summer (June-August) 2017. Maps are eastern US OA (a), sum of pNO_3_ and pNH_4_ (b), isoprene SOA (c) and PM_2.5_ (d) from the difference in GEOS-Chem simulations with and without end-use emissions and, for pNO_3_, pNH_4_ and PM_2.5_, corrected for model biases using the surface network observations (see Section 3.2 for details).

Figures 3 and 4 show the influence of end-use activities on gas-phase (Figure 3) and particle-phase (Figure 4) pollutants obtained as the difference between model simulations with and without end-use emissions and corrected for model biases in pNO_3_, pNH_4_ and PM_2.5_ (Section 3.2). End-use activities make a large contribution to summertime mean NO_*x*_ of up to 20 ppb. More than 90% of this is NO_2_ that is mostly collocated with major cities and highways (Figure 3a). Even though the majority of NO_*x*_ is emitted as NO, the end-use activity NO concentration is small (0.12 ppb on average), as NO rapidly converts to NO_2_, maintaining eastern US mean NO/NO_2_ ratios at ∼9% in both simulations. As a result of the small contribution of NO to NO_*x*_, the shift in isoprene from oxidation via the NO pathway to oxidation via the HO_2_ pathway is small. In the absence of end-use emissions, 42% of isoprene is oxidized by NO and 30% by HO_2_. The proportion is 49:27 NO:HO_2_ with end-use emissions. The additional branching attributed collectively to the NO and HO_2_ pathway with end-use activities is because the less prevalent isomerization^92,93^ branch declines with end-use emissions. The modest response of the branching ratios to end-use activities is fairly consistent across the region, though these differ between the north (55:25 NO:HO_2_ without end-use, 62:22 NO:HO_2_ with) and south (34:33 NO:HO_2_ without end-use, 40:30 NO:HO_2_ with) due to greater NO_*x*_ abundance in the north.

Large end-use activity enhancements in HCHO in the southeast and along the northeast coast (Figure 3b) result from higher and more prompt yields of HCHO (Figure 3b) via the NO isoprene oxidation pathway than the HO_2_ pathway.^40,94^ We estimate that end-use activity NO_*x*_ emissions increase isoprene oxidation HCHO yields by 8-10% over the locations in Figure 3b, exceeding 400 ppt from interpolation of reported GEOS-Chem chemical mechanism HCHO yields at 1 ppb NO_*x*_ (∼1.9 moles HCHO per mole isoprene) and at 0.1 ppb NO_*x*_ (∼1.6 mol mol^–1^).^40^ Secondary production of HCHO attributable to end-use activities exceeds 1 ppb in and around Atlanta, coincident with the NO_2_ hotspot in Figure 3a and where interaction of biogenic and anthropogenic emissions is well-known to degrade air quality.^95−97^ The enhanced yields of HCHO and other small reactive oxygenated VOCs from isoprene oxidation also contribute to MDA8 O_3_ in the region, as O_3_ production is limited by availability of VOCs.^98^ Overall, end-use emissions contribute at least 5 ppb MDA8 O_3_ in most (∼80%) of the eastern US and >14 ppb across a large swath of eastern US from north Georgia extending as far north as southern New England (Figure 3c). The role of end-use primary HCHO (12 Gg) in the enhancement in Figure 3b and in forming MDA8 O_3_ is minimal in comparison to secondary HCHO, as emissions of primary HCHO are limited to major cities. These primary HCHO emissions are evident as faint (<300 ppt) isolated enhancements in cities such as Pittsburgh, Detroit and Chicago located north of the southeast US biogenic HCHO enhancement.

The contribution of end-use activities to OA across the eastern US exceeds 2 μg m^–3^ along the northeast corridor and in large cities (Figure 4a). Most of this is due to anthropogenic OA from primary and precursor VOCs emissions from vehicles and from VCPs concentrated in cities. Eastern US mean end-use POA is 0.11 μg m^–3^ and anthropogenic SOA is 0.47 μg m^–3^. It is not possible to quantify the influence of individual VOCs or source types on anthropogenic SOA, as CO emissions are used to estimate anthropogenic SOA in GEOS-Chem (Section 2.1). End-use activities account for most of the anthropogenic CO emissions (Figure 2), resulting in a large decline in OA. Use of CO as a proxy for SOA formation is standard^99,100^ and defensible,^63^ given the limited detailed knowledge of VOCs emissions and reaction pathways forming SOA,^101^ challenges representing these in models without large computational burden, and the major (∼80%) contribution of end-use activity emissions of known SOA precursors such as alkanes and toluene to total anthropogenic emissions in the US NEI.

End-use NO_*x*_ increases the abundance of acidic pNO_3_ that in turn promotes uptake of ammonia, mainly from agriculture,^102,103^ forming pNH_4_ (Figure 4b). As a result, pNO_3_ and pNH_4_ are typically collocated. The corrected pNO_3_ and pNH_4_ (Section 3.2) are similar in magnitude to that of end-use pSO_4_ (∼0.1-0.2 μg m^–3^) (not shown), but the spatial distribution of end-use pSO_4_ differs. The greatest contribution of end-use activities to pNO_3_ and pNH_4_ of up to 0.6 μg m^–3^ is in the northeast.

The end-use activity effect on isoprene SOA is mixed. Influence of end-use NO_*x*_ emissions on the oxidative fate of isoprene (Section 3.2) suppresses formation of the SOA precursors IEPOX and C_5_-LVOCs and promotes formation of the SOA precursor glyoxal, though the SOA yields of this precursor are uncertain.^36^ End-use pSO_4_ enhances isoprene SOA formation by increasing the abundance of acidic aqueous-phase aerosols. The net effect is suppression of isoprene SOA formation, though the effect is small; 0.02-0.03 μg m^–3^ in most of the US and 0.06-0.08 μg m^–3^ in large cities in the southeast (Figure 4c). End-use BC (not shown), a potent short-lived climate forcer, is on average about 0.1 μg m^–3^ in the eastern US and 0.3-0.4 μg m^–3^ in large cities.

The net effect of end-use activities on PM_2.5_ (Figure 4d) is a 1.5 μg m^–3^ contribution in most of the eastern US and >3 μg m^–3^ contribution in cities and along the northeast coast. Anthropogenic OA makes the greatest and most widespread contribution to end-use PM_2.5_ concentrations, followed by BC and the inorganic secondary aerosols pSO_4_, pNO_3_, and pNH_4_.

### 3.4 Pathways of Influence of Oil and Gas End-Use on Atmospheric Composition

**Figure 5:**
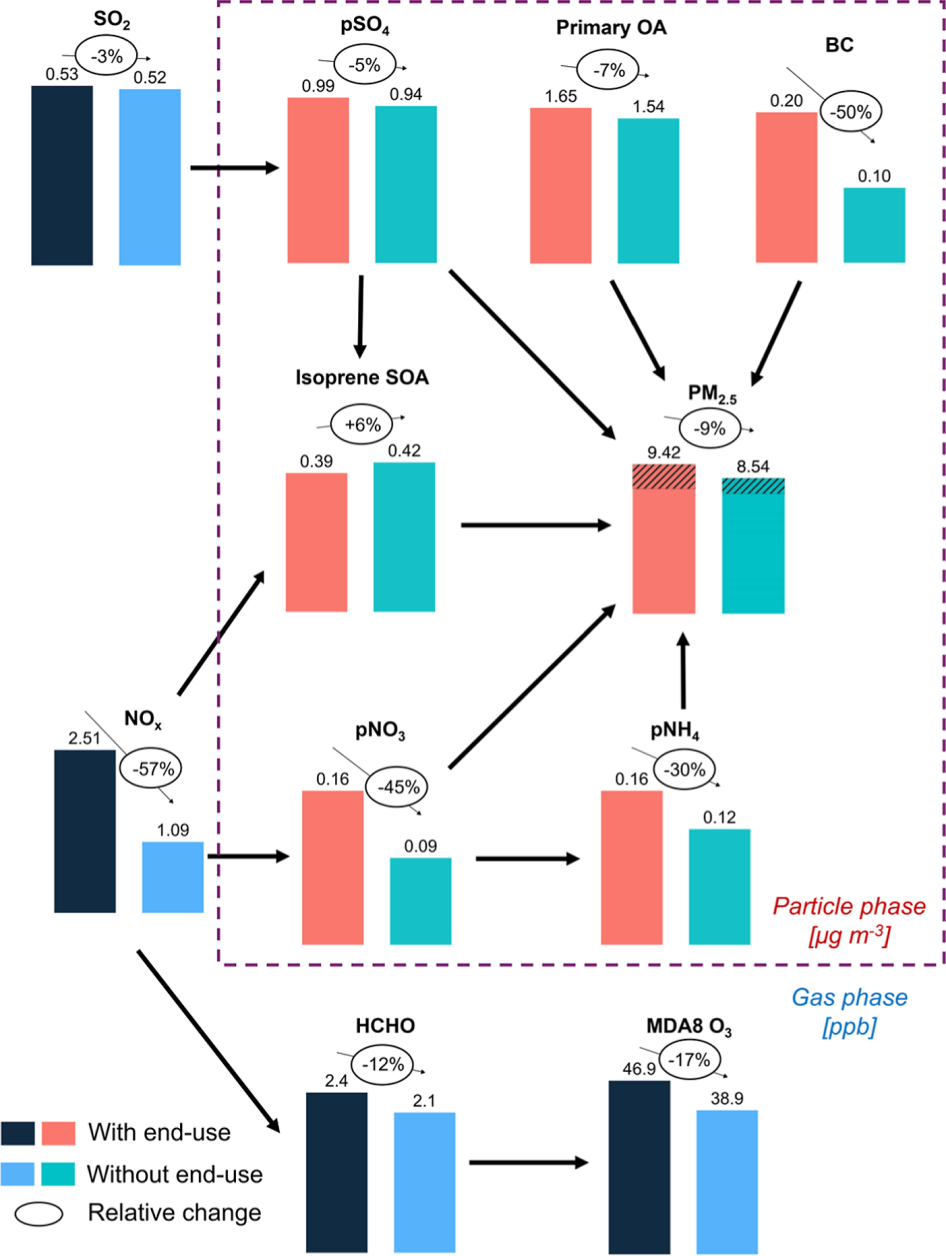
Influence of oil and gas end-use on air pollutants in eastern US in summer (June-August) 2017. Bars and values above each bar indicate summer mean concentrations with and without end-use activity emissions and values in bubbles above bars are percent changes attributable to end-use activities. The dashed box distinguishes gas- and aerosol-phase pollutants. Hatched portion of PM_2.5_ is anthropogenic SOA parametrized in GEOS-Chem using CO emissions as a proxy (Section 2.1). A small contribution of end-use pSO_4_, pNO_3_ and pNH_4_ is from primary sources. With-end-use simulated summer mean concentrations of pNO_3_ and pNH_4_ are corrected for model biases by dividing by model NMBs in Figure 2. Without-end-use means are recomputed using the corrected with-end-use means and end-use attributable means (correction to latter detailed in Section 3.2). PM_2.5_ for both simulations is recomputed using the corrected pNO_3_ and pNH_4_.

Figure 5 summarizes the primary and secondary routes of influence of end-use activities on eastern US mean concentrations of the health-damaging pollutants NO_2_, PM_2.5_, HCHO, and MDA8 O_3_. The direct effect of end-use activity emissions (Figure 1) on ambient concentrations of these and precursors to these pollutants is 1.4 ppb NO_*x*_, 17 ppt SO_2_, and 0.1 μg m^–3^ each of BC and primary OA. As pSO_4_, pNO_3_, pNH_4_, and BC loss processes are the same, we use the BC end-use emissions-to-concentration ratio of 220 Gg (μg m^–3^)^−1^ to estimate that 1.7 Gg primary end-use pSO_4_ accounts for ∼16% of end-use pSO_4_, 0.32 Gg primary end-use pNO_3_ for only 2% of end-use pNO_3_, and 0.69 Gg primary end-use pNH_4_ for 6% of end-use pNH_4_. Primary formation of HCHO is prevalent in urban areas (Section 3.3), but even in cities with relatively large anthropogenic HCHO sources attributable to end-use activities, most anthropogenic HCHO is secondary, from oxidation of VOCs.^104^

Secondary effects include the influence of end-use NO_*x*_ on the oxidative fate of isoprene that suppresses isoprene SOA formation by just 0.02 μg m^–3^, but promotes formation of 300 ppt HCHO via the prompt and high-HCHO-yield NO oxidation pathway (Section 3.3). The greater yields of HCHO and other associated small reactive oxygenated VOCs also enhance O_3_ formation, contributing to 8 ppb MDA8 O_3_. Other major secondary processes that affect end-use PM_2.5_ is SOA sourced from anthropogenic VOCs, pNO_3_ from oxidation of NO_*x*_, pSO_4_ from oxidation of SO_2_, and pNH_4_ from reversible partitioning of NH_3_ to neutralize acidic aerosols. The largest contributor to end-use PM_2.5_ is SOA (0.47 μg m^–3^). A meagre 17 ppt SO_2_ from end-use activities has an outsized effect on pSO_4_ (0.04 μg m^–3^) compared to a similar effect of 1.42 ppb end-use NO_*x*_ (90% NO_2_) on pNO_3_ (0.07 μg m^–3^) that in turn promotes formation of a similar amount of pNH_4_ (0.05 μg m^–3^). PM_2.5_ components are mostly enhanced over urban areas and the northeast corridor, except for the enhancement in pNO_3_ and pNH_4_ that occurs over the agriculturally intensive corn belt (Figure 4b), as most US NH_3_ emissions are from agricultural activity^102,103^ that is well-known to exacerbate PM_2.5_ formed from other sector activities.^105,106^

Overall, end-use PM_2.5_ from primary and secondary processes is 0.88 μg m^–3^. This is ∼9% of eastern US PM_2.5_, though this is conservative, as GEOS-Chem PM_2.5_ has a positive bias in background PM_2.5_ that is likely due to a model overestimate in dust (Supporting Information Text S1 ). If we correct for our estimated systematic model bias of ∼2.8 μg m^–3^, the contribution increases to ∼13%. The influence estimates from this work are not directly comparable to previous studies, as past studies have either lumped together oil and gas end-use with coal, focused on a subset of end-use activities such as power generation, did not consider the primary emissions of air pollutant precursors to disentangle the secondary effects, or conducted annual assessments for the US that dampens the influence of summertime peak biogenic emissions on atmospheric chemistry and air pollution.

Public health concerns that our results raise include long-term exposure to traffic-related NO_2_ that has been linked to the increase in childhood asthma incidences by 1.26% for every 10 ppb increase in NO_2_, even at low concentrations (∼2 ppb) that end-use NO_2_ far exceeds in cities in the eastern US (Figure 3a).^5,15,107^ Long-term exposure to PM_2.5_ at concentrations typical of the eastern US (10-12 μg m^–3^; Figure S2a) increase the risk of all-cause premature mortality by 1% with a 1 μg m^–3^ increase in annual mean PM_2.5_, a threshold exceeded by almost half (48% area) the eastern US in summer (Figure 4d).^108^ Exposure to 0.3 ppb of end-use HCHO over a person’s statistical lifespan is associated with an increased cancer risk of ∼5-6 in a million,^109^ so would exceed 10 in a million in the isoprene-rich southeast US where end-use HCHO is at least 0.6 ppb (Figure 3b). End-use activities increase peak summer season MDA8 O_3_ well beyond the safe threshold of ∼32 ppb (Figure 3c), increasing the risk of premature mortality from chronic respiratory diseases by 6% for every 10 ppb increase in MDA8 O_3_.^110,111^ End-use MDA8 O_3_ in about 20% of eastern US exceeds 10 ppb. There is potential for substantial public health benefits from decline in oil and gas consumption, though only to the extent that cleaner alternatives adopted have limited unintended consequences on air quality. In that sense, our estimates represent upper-bounds of air quality improvements that could be achieved from policies that promote adoption of cleaner alternatives.

There are many emissions and model improvements that are needed to further refine understanding of the implications of oil and gas end-use activity emissions on summertime air quality. These include: (1) guidance from the US EPA on characterizing NEI “Other PM_2.5_” emissions, (2) addressing the cause of the model bias in pNO_3_ and pNH_4_ to avoid reliance on dense ground-based monitoring networks to derive correction factors and to utilize evidence of variable toxicity of individual PM_2.5_ components in burden of disease studies,^112,113^ (3) explicitly represent pathways from the suite of VOCs to anthropogenic SOA, as is being developed for VCPs,^13^ and (4) mechanistic representation of gas- and aerosol-phase chemistry of biogenic VOCs like monoterpenes and sesquiterpenes and the complex interactions between isoprene SOA and monoterpene SOA.^114^

Nonetheless, our results suggest that reducing the use of processed and unprocessed oil and natural gas in the eastern US has the potential to significantly benefit public health by simultaneously decreasing concentrations of multiple harmful pollutants emitted directly as POA, generated as anthropogenic SOA, formed as O_3_ and HCHO from large influence of end-use NO_*x*_ on the oxidative fate of biogenic isoprene, and formed as pNH_4_ from partitioning of agricultural sources of NH_3_. These effects are examined for the summertime in the eastern US, but are likely to persist year-round in the tropics where fossil fuels are already a dominant energy source,^115^ where oil and gas demand is growing rapidly, particularly across Africa,^116,117^ and where effective environmental policies are lacking and barriers to adopting cleaner sources of energy persist. There are large sources of uncertainty and measurement and data collection gaps in the tropics that need to be addressed to similarly determine the complex pathways degrading air quality from interaction between oil and gas end-use activity and isoprene emissions.

## Data Availability

The gridded hourly July 2017 end-use emissions generated using NEI SCC codes are available for download from the NOAA data portal (https://csl.noaa.gov/groups/csl7/measurements/2020covid-aqs/emissions/).

## Supporting Information Available

Supporting Information includes the detailed evaluation of the GEOS-Chem model and a tabulated list of activities classified as oil and gas end-use (PDF)

## References

[ref1] Anenberg S. C.; Miller J.; Minjares R.; Du L.; Henze D. K.; Lacey F.; Malley C. S.; Emberson L.; Franco V.; Klimont Z.; Heyes C. Impacts and mitigation of excess diesel-related NOx emissions in 11 major vehicle markets. Nature 2017, 545 (7655), 467–471. 10.1038/nature22086. 28505629

[ref2] Colvile R. N.; Hutchinson E. J.; Mindell J. S.; Warren R. F. The transport sector as a source of air pollution. Atmos. Environ. 2001, 35 (9), 1537–1565. 10.1016/S1352-2310(00)00551-3.

[ref3] Zhang R. N.; Li H.; Chen T. Q.; Hou B. D. How does natural gas consumption affect human health? Empirical evidence from China. J. Cleaner Prod. 2021, 320, 128795 10.1016/j.jclepro.2021.128795.

[ref4] Mayfield E. N.; Cohon J. L.; Muller N. Z.; Azevedo I. M. L.; Robinson A. L. Cumulative environmental and employment impacts of the shale gas boom. Nat. Sustain. 2019, 2 (12), 1122–1131. 10.1038/s41893-019-0420-1. 31844682 PMC6914251

[ref5] Achakulwisut P.; Brauer M.; Hystad P.; Anenberg S. C. Global, national, and urban burdens of paediatric asthma incidence attributable to ambient NO_2_ pollution: estimates from global datasets. Lancet Planet Health 2019, 3 (4), E166–E178. 10.1016/S2542-5196(19)30046-4. 30981709

[ref6] US Dept of Energy. U.S. OIL AND NATURAL GAS: Providing Energy Security and Supporting Our Quality of Life. 2020. https://www.energy.gov/sites/prod/files/2020/10/f79/NaturalGasBenefitsReport.pdf. accessed 3 June 2024.

[ref7] US EIA. U.S. energy facts explained, 2022. https://www.eia.gov/energyexplained/us-energy-facts/. accessed 3 March 2024.

[ref8] Harkins C.; McDonald B. C.; Henze D. K.; Wiedinmyer C. A fuel-based method for updating mobile source emissions during the COVID-19 pandemic. Environ. Res. Lett. 2021, 16 (6), 065018 10.1088/1748-9326/ac0660.

[ref9] Fine P. M.; Sioutas C.; Solomon P. A. Secondary particulate matter in the United States: Insights from the particulate matter supersites program and related studies. J. Air Waste Manage 2008, 58 (2), 234–253. 10.3155/1047-3289.58.2.234. 18318339

[ref10] Park R. J.; Jacob D. J.; Field B. D.; Yantosca R. M.; Chin M. Natural and transboundary pollution influences on sulfate-nitrate-ammonium aerosols in the United States: Implications for policy. J. Geophys. Res.: Atmos. 2004, 109( (D15), ). 10.1029/2003JD004473.

[ref11] McDonald B. C.; de Gouw J. A.; Gilman J. B.; Jathar S. H.; Akherati A.; Cappa C. D.; Jimenez J. L.; Lee-Taylor J.; Hayes P. L.; McKeen S. A.; Cui Y. Y.; Kim S. W.; Gentner D. R.; Isaacman-VanWertz G.; Goldstein A. H.; Harley R. A.; Frost G. J.; Roberts J. M.; Ryerson T. B.; Trainer M. Volatile chemical products emerging as largest petrochemical source of urban organic emissions. Science 2018, 359 (6377), 760–764. 10.1126/science.aaq0524. 29449485

[ref12] Seltzer K. M.; Pennington E.; Rao V.; Murphy B. N.; Strum M.; Isaacs K. K.; Pye H. O. T. Reactive organic carbon emissions from volatile chemical products. Atmos. Chem. Phys. 2021, 21 (6), 5079–5100. 10.5194/acp-21-5079-2021. 34122530 PMC8193795

[ref13] Pennington E. A.; Seltzer K. M.; Murphy B. N.; Qin M. M.; Seinfeld J. H.; Pye H. O. T. Modeling secondary organic aerosol formation from volatile chemical products. Atmos. Chem. Phys. 2021, 21 (24), 18247–18261. 10.5194/acp-21-18247-2021. 35087576 PMC8788583

[ref14] Sasidharan S.; He Y. C.; Akherati A.; Li Q.; Li W. H.; Cocker D.; McDonald B. C.; Coggon M. M.; Seltzer K. M.; Pye H. O. T.; Pierce J. R.; Jathar S. H. Secondary Organic Aerosol Formation from Volatile Chemical Product Emissions: Model Parameters and Contributions to Anthropogenic Aerosol. Environ. Sci. Technol. 2023, 57 (32), 11891–11902. 10.1021/acs.est.3c00683. 37527511 PMC11610419

[ref15] Khreis H.; Kelly C.; Tate J.; Parslow R.; Lucas K.; Nieuwenhuijsen M. Exposure to traffic-related air pollution and risk of development of childhood asthma: A systematic review and meta-analysis. Environ. Int. 2017, 100, 1–31. 10.1016/j.envint.2016.11.012. 27881237

[ref16] Brauer M.; Brook J. R.; Crouse D.; Erickson A.; Hystad P.; Li C.; Martin R.; Meng J.; Tjepkema M.; van Donkelaar A.; Christidis T.; Pinault L. L.; Yuchi W.; Weagle C.; Weichenthal S.; Burnett R.; Burnett R. T. Air pollution impacts at very low levels: Shape of the concentration-mortality relationship in a large population-based Canadian cohort. Res. Rep. - Health Eff. Inst. 2022, 2022 (1), 1–91. 10.1289/isee.2022.p-0237.

[ref17] Burnett R.; Chen H.; Szyszkowicz M.; Fann N.; Hubbell B.; Pope C. A.; Apte J. S.; Brauer M.; Cohen A.; Weichenthal S.; Coggins J.; Di Q.; Brunekreef B.; Frostad J.; Lim S. S.; Kan H. D.; Walker K. D.; Thurston G. D.; Hayes R. B.; Lim C. C.; Turner M. C.; Jerrett M.; Krewski D.; Gapstur S. M.; Diver W. R.; Ostro B.; Goldberg D.; Crouse D. L.; Martin R. V.; Peters P.; Pinault L.; Tjepkema M.; van Donkelaar A.; Villeneuve P. J.; Miller A. B.; Yin P.; Zhou M. G.; Wang L. J.; Janssen N. A. H.; Marra M.; Atkinson R. W.; Tsang H.; Quoc Thach T.; Cannon J. B.; Allen R. T.; Hart J. E.; Laden F.; Cesaroni G.; Forastiere F.; Weinmayr G.; Jaensch A.; Nagel G.; Concin H.; Spadaro J. V. Global estimates of mortality associated with long-term exposure to outdoor fine particulate matter. Proc. Natl. Acad. Sci. U.S.A. 2018, 115 (38), 9592–9597. 10.1073/pnas.1803222115. 30181279 PMC6156628

[ref18] Vohra K.; Vodonos A.; Schwartz J.; Marais E. A.; Sulprizio M. P.; Mickley L. J. Global mortality from outdoor fine particle pollution generated by fossil fuel combustion: Results from GEOS-Chem. Environ. Res. 2021, 195, 110754 10.1016/j.envres.2021.110754. 33577774

[ref19] Buonocore J. J.; Reka S.; Yang D.; Chang C.; Roy A.; Thompson T.; Lyon D.; McVay R.; Michanowicz D.; Arunachalam S. Air pollution and health impacts of oil & gas production in the United States. Environ. Res. Health 2023, 1 (2), 021006 10.1088/2752-5309/acc886.

[ref20] Fann N.; Baker K. R.; Chan E. A. W.; Eyth A.; Macpherson A.; Miller E.; Snyder J. Assessing Human Health PM_2.5_ and Ozone Impacts from US Oil and Natural Gas Sector Emissions in 2025. Environ. Sci. Technol. 2018, 52 (15), 8095–8103. 10.1021/acs.est.8b02050. 30004688 PMC6718951

[ref21] Allen D. T. Emissions from oil and gas operations in the United States and their air quality implications. J. Air Waste Manage 2016, 66 (6), 549–575. 10.1080/10962247.2016.1171263. 27249104

[ref22] Field R. A.; Soltis J.; Murphy S. Air quality concerns of unconventional oil and natural gas production. Environ. Sci.: Processes Impacts 2014, 16 (5), 954–969. 10.1039/C4EM00081A. 24699994

[ref23] Thompson T. M.; Shepherd D.; Stacy A.; Barna M. G.; Schichtel B. A. Modeling to Evaluate Contribution of Oil and Gas Emissions to Air Pollution. J. Air Waste Manage 2017, 67 (4), 445–461. 10.1080/10962247.2016.1251508. 27819534

[ref24] Vohra K.; Marais E. A.; Bloss W. J.; Schwartz J.; Mickley L. J.; Van Damme M.; Clarisse L.; Coheur P. F. Rapid rise in premature mortality due to anthropogenic air pollution in fast-growing tropical cities from 2005 to 2018. Sci. Adv. 2022, 8( (14), ). 10.1126/sciadv.abm4435. PMC8993110 35394832

[ref25] Hoornweg D.; Pope K. Population predictions for the world’s largest cities in the 21st century. Environ. Urban 2017, 29 (1), 195–216. 10.1177/0956247816663557.

[ref26] Archer C. L.; Cervone G.; Golbazi M.; Al Fahel N.; Hultquist C. Changes in air quality and human mobility in the USA during the COVID-19 pandemic. Bull. Atmos. Sci. Technol. 2020, 1 (3–4), 491–514. 10.1007/s42865-020-00019-0. 38624442 PMC7586872

[ref27] Goldberg D. L.; Anenberg S. C.; Griffin D.; McLinden C. A.; Lu Z. F.; Streets D. G. Disentangling the Impact of the COVID-19 Lockdowns on Urban NO2 From Natural Variability. Geophys. Res. Lett. 2020, 47( (17), ). 10.1029/2020GL089269. PMC7461033 32904906

[ref28] Berman J. D.; Ebisu K. Changes in U.S. air pollution during the COVID-19 pandemic. Sci. Total Environ. 2020, 739, 139864 10.1016/j.scitotenv.2020.139864. 32512381 PMC7442629

[ref29] He J.; Harkins C.; O’Dell K.; Li M.; Francoeur C.; Aikin K. C.; Anenberg S.; Baker B.; Brown S. S.; Coggon M. M.; Frost G. J.; Gilman J. B.; Kondragunta S.; Lamplugh A.; Lyu C.; Moon Z.; Pierce B. R.; Schwantes R. H.; Stockwell C. E.; Warneke C.; Yang K.; Nowlan C. R.; González Abad G.; McDonald B. C. COVID-19 perturbation on US air quality and human health impact assessment. PNAS Nexus 2023, 3 (1), pgad483 10.1093/pnasnexus/pgad483. PMC10785034 38222466

[ref30] Hammer M. S.; van Donkelaar A.; Martin R. V.; McDuffie E. E.; Lyapustin A.; Sayer A. M.; Hsu N. C.; Levy R. C.; Garay M. J.; Kalashnikova O. V.; Kahn R. A. Effects of COVID-19 lockdowns on fine particulate matter concentrations. Sci. Adv. 2021, 7( (26), ). 10.1126/sciadv.abg7670. PMC8221629 34162552

[ref31] Ghahremanloo M.; Lops Y.; Choi Y.; Jung J.; Mousavinezhad S.; Hammond D. A comprehensive study of the COVID-19 impact on PM2.5 levels over the contiguous United States: A deep learning approach. Atmos. Environ. 2022, 272, 118944 10.1016/j.atmosenv.2022.118944. PMC8758197 35043042

[ref32] Bar S.; Parida B. R.; Mandal S. P.; Pandey A. C.; Kumar N.; Mishra B. Impacts of partial to complete COVID-19 lockdown on NO2 and PM2.5 levels in major urban cities of Europe and USA. Cities 2021, 117, 103308 10.1016/j.cities.2021.103308. 34127873 PMC8189822

[ref33] Campbell P. C.; Tong D.; Tang Y. H.; Baker B.; Lee P.; Saylor R.; Stein A.; Ma S. Q.; Lamsal L.; Qu Z. Impacts of the COVID-19 economic slowdown on ozone pollution in the U. S. Atmos. Environ. 2021, 264, 118713 10.1016/j.atmosenv.2021.118713. PMC8430042 34522157

[ref34] Rieder H. E.; Fiore A. M.; Polvani L. M.; Lamarque J. F.; Fang Y. Changes in the frequency and return level of high ozone pollution events over the eastern United States following emission controls. Environ. Res. Lett. 2013, 8 (1), 014012 10.1088/1748-9326/8/1/014012.

[ref35] Guenther A. B.; Jiang X.; Heald C. L.; Sakulyanontvittaya T.; Duhl T.; Emmons L. K.; Wang X. The Model of Emissions of Gases and Aerosols from Nature version 2.1 (MEGAN2.1): an extended and updated framework for modeling biogenic emissions. Geosci. Model Dev. 2012, 5 (6), 1471–1492. 10.5194/gmd-5-1471-2012.

[ref36] Marais E. A.; Jacob D. J.; Jimenez J. L.; Campuzano-Jost P.; Day D. A.; Hu W.; Krechmer J.; Zhu L.; Kim P. S.; Miller C. C.; Fisher J. A.; Travis K.; Yu K.; Hanisco T. F.; Wolfe G. M.; Arkinson H. L.; Pye H. O. T.; Froyd K. D.; Liao J.; McNeill V. F. Aqueous-phase mechanism for secondary organic aerosol formation from isoprene: application to the southeast United States and co-benefit of SO2 emission controls. Atmos. Chem. Phys. 2016, 16 (3), 1603–1618. 10.5194/acp-16-1603-2016. 32742280 PMC7394309

[ref37] Mettke P.; Brüggemann M.; Mutzel A.; Gräfe R.; Herrmann H. Secondary Organic Aerosol (SOA) through Uptake of Isoprene Hydroxy Hydroperoxides (ISOPOOH) and its Oxidation Products. ACS Earth Space Chem. 2023, 7 (5), 1025–1037. 10.1021/acsearthspacechem.2c00385.

[ref38] Hu W. W.; Palm B. B.; Day D. A.; Campuzano-Jost P.; Krechmer J. E.; Peng Z.; de Sá S. S.; Martin S. T.; Alexander M. L.; Baumann K.; Hacker L.; Kiendler-Scharr A.; Koss A. R.; de Gouw J. A.; Goldstein A. H.; Seco R.; Sjostedt S. J.; Park J. H.; Guenther A. B.; Kim S.; Canonaco F.; Prévôt A. S. H.; Brune W. H.; Jimenez J. L. Volatility and lifetime against OH heterogeneous reaction of ambient isoprene-epoxydiols-derived secondary organic aerosol (IEPOX-SOA). Atmos. Chem. Phys. 2016, 16 (18), 11563–11580. 10.5194/acp-16-11563-2016.

[ref39] Wolfe G. M.; Kaiser J.; Hanisco T. F.; Keutsch F. N.; de Gouw J. A.; Gilman J. B.; Graus M.; Hatch C. D.; Holloway J.; Horowitz L. W.; Lee B. H.; Lerner B. M.; Lopez-Hilifiker F.; Mao J.; Marvin M. R.; Peischl J.; Pollack I. B.; Roberts J. M.; Ryerson T. B.; Thornton J. A.; Veres P. R.; Warneke C. Formaldehyde production from isoprene oxidation across NO_*x*_ regimes. Atmos. Chem. Phys. 2016, 16 (4), 2597–2610. 10.5194/acp-16-2597-2016. 29619046 PMC5879783

[ref40] Marais E. A.; Jacob D. J.; Kurosu T. P.; Chance K.; Murphy J. G.; Reeves C.; Mills G.; Casadio S.; Millet D. B.; Barkley M. P.; Paulot F.; Mao J. Isoprene emissions in Africa inferred from OMI observations of formaldehyde columns. Atmos. Chem. Phys. 2012, 12 (14), 6219–6235. 10.5194/acp-12-6219-2012. 33688332 PMC7939075

[ref41] Krechmer J. E.; Coggon M. M.; Massoli P.; Nguyen T. B.; Crounse J. D.; Hu W. W.; Day D. A.; Tyndall G. S.; Henze D. K.; Rivera-Rios J. C.; Nowak J. B.; Kimmel J. R.; Mauldin R. L.; Stark H.; Jayne J. T.; Sipilä M.; Junninen H.; St Clair J. M.; Zhang X.; Feiner P. A.; Zhang L.; Miller D. O.; Brune W. H.; Keutsch F. N.; Wennberg P. O.; Seinfeld J. H.; Worsnop D. R.; Jimenez J. L.; Canagaratna M. R. Formation of Low Volatility Organic Compounds and Secondary Organic Aerosol from Isoprene Hydroxyhydroperoxide Low-NO Oxidation. Environ. Sci. Technol. 2015, 49 (17), 10330–10339. 10.1021/acs.est.5b02031. 26207427

[ref42] Bates K. H.; Jacob D. J. A new model mechanism for atmospheric oxidation of isoprene: global effects on oxidants, nitrogen oxides, organic products, and secondary organic aerosol. Atmos. Chem. Phys. 2019, 19 (14), 9613–9640. 10.5194/acp-19-9613-2019.

[ref43] Budisulistiorini S. H.; Li X.; Bairai S. T.; Renfro J.; Liu Y.; Liu Y. J.; McKinney K. A.; Martin S. T.; McNeill V. F.; Pye H. O. T.; Nenes A.; Neff M. E.; Stone E. A.; Mueller S.; Knote C.; Shaw S. L.; Zhang Z.; Gold A.; Surratt J. D. Examining the effects of anthropogenic emissions on isoprene-derived secondary organic aerosol formation during the 2013 Southern Oxidant and Aerosol Study (SOAS) at the Look Rock, Tennessee ground site. Atmos. Chem. Phys. 2015, 15 (15), 8871–8888. 10.5194/acp-15-8871-2015.

[ref44] Weber R. J.; Guo H. Y.; Russell A. G.; Nenes A. High aerosol acidity despite declining atmospheric sulfate concentrations over the past 15 years. Nat. Geosci. 2016, 9 (4), 282–285. 10.1038/ngeo2665.

[ref45] Lawal A. S.; Guan X. B.; Liu C.; Henneman L. R. F.; Vasilakos P.; Bhogineni V.; Weber R. J.; Nenes A.; Russell A. G. Linked Response of Aerosol Acidity and Ammonia to SO_2_ and NO_x_ Emissions Reductions in the United States. Environ. Sci. Technol. 2018, 52 (17), 9861–9873. 10.1021/acs.est.8b00711. 30032604

[ref46] Chen Y. L.; Shen H. Z.; Russell A. G. Current and Future Responses of Aerosol pH and Composition in the US to Declining SO_2_ Emissions and Increasing NH_3_ Emissions. Environ. Sci. Technol. 2019, 53 (16), 9646–9655. 10.1021/acs.est.9b02005. 31369250

[ref47] Nopmongcol U.; Beardsley R.; Kumar N.; Knipping E.; Yarwood G. Changes in United States deposition of nitrogen and sulfur compounds over five decades from 1970 to 2020. Atmos. Environ. 2019, 209, 144–151. 10.1016/j.atmosenv.2019.04.018.

[ref48] McDonald B. C.; McKeen S. A.; Cui Y. Y.; Ahmadov R.; Kim S. W.; Frost G. J.; Pollack I. B.; Peischl J.; Ryerson T. B.; Holloway J. S.; Graus M.; Warneke C.; Gilman J. B.; de Gouw J. A.; Kaiser J.; Keutsch F. N.; Hanisco T. F.; Wolfe G. M.; Trainer M. Modeling Ozone in the Eastern US using a Fuel-Based Mobile Source Emissions Inventory. Environ. Sci. Technol. 2018, 52 (13), 7360–7370. 10.1021/acs.est.8b00778. 29870662

[ref49] Li M.; McDonald B. C.; McKeen S. A.; Eskes H.; Levelt P.; Francoeur C.; Harkins C.; He J.; Barth M.; Henze D. K.; Bela M. M.; Trainer M.; de Gouw J. A.; Frost G. J. Assessment of Updated Fuel-Based Emissions Inventories Over the Contiguous United States Using TROPOMI NO_2_ Retrievals. J. Geophys. Res.: Atmos. 2021, 126( (24), ). 10.1029/2021JD035484.

[ref50] Yu K. A.; McDonald B. C.; Harley R. A. Evaluation of Nitrogen Oxide Emission Inventories and Trends for On-Road Gasoline and Diesel Vehicles. Environ. Sci. Technol. 2021, 55 (10), 6655–6664. 10.1021/acs.est.1c00586. 33951912

[ref51] Simone N. W.; Stettler M. E. J.; Barrett S. R. H. Rapid estimation of global civil aviation emissions with uncertainty quantification. Transp. Res. D Trans. Environ. 2013, 25, 33–41. 10.1016/j.trd.2013.07.001.

[ref52] Stettler M. E. J.; Eastham S.; Barrett S. R. H. Air quality and public health impacts of UK airports. Part I: Emissions. Atmos. Environ. 2011, 45 (31), 5415–5424. 10.1016/j.atmosenv.2011.07.012.

[ref53] Hudman R. C.; Moore N. E.; Mebust A. K.; Martin R. V.; Russell A. R.; Valin L. C.; Cohen R. C. Steps towards a mechanistic model of global soil nitric oxide emissions: implementation and space based-constraints. Atmos. Chem. Phys. 2012, 12 (16), 7779–7795. 10.5194/acp-12-7779-2012.

[ref54] Jaegle L.; Quinn P. K.; Bates T. S.; Alexander B.; Lin J. T. Global distribution of sea salt aerosols: new constraints from in situ and remote sensing observations. Atmos. Chem. Phys. 2011, 11 (7), 3137–3157. 10.5194/acp-11-3137-2011.

[ref55] Meng J.; Martin R. V.; Ginoux P.; Hammer M.; Sulprizio M. P.; Ridley D. A.; van Donkelaar A. Grid-independent high-resolution dust emissions (v1.0) for chemical transport models: application to GEOS-Chem. (12.5.0). Geosci. Model Dev. 2021, 14 (7), 4249–4260. 10.5194/gmd-14-4249-2021.

[ref56] Murray L. T. Lightning NO_*x*_ and Impacts on Air Quality. Curr. Pollut. Rep. 2016, 2 (2), 115–133. 10.1007/s40726-016-0031-7.

[ref57] Fountoukis C.; Nenes A. ISORROPIA II:: a computationally efficient thermodynamic equilibrium model for K^+^-Ca^2+^-Mg^2+^-NH_4_^+^-Na^+^-SO_4_^2-^-NO_3_^–^-Cl^–^-H_2_O aerosols. Atmos. Chem. Phys. 2007, 7 (17), 4639–4659. 10.5194/acp-7-4639-2007.

[ref58] Luo G.; Yu F. Q.; Schwab J. Revised treatment of wet scavenging processes dramatically improves GEOS-Chem. 12.0.0 simulations of surface nitric acid, nitrate, and ammonium over the United States. Geosci. Model Dev. 2019, 12 (8), 3439–3447. 10.5194/gmd-12-3439-2019.

[ref59] Tie X. X.; Madronich S.; Walters S.; Edwards D. P.; Ginoux P.; Mahowald N.; Zhang R. Y.; Lou C.; Brasseur G. Assessment of the global impact of aerosols on tropospheric oxidants. J. Geophys. Res.: Atmos. 2005, 110( (D3), ). 10.1029/2004JD005359.

[ref60] Cooke W. F.; Liousse C.; Cachier H.; Feichter J. Construction of a 1° × 1° fossil fuel emission data set for carbonaceous aerosol and implementation and radiative impact in the ECHAM4 model. J. Geophys. Res.: Atmos. 1999, 104 (D18), 22137–22162. 10.1029/1999jd900187.

[ref61] Chin M.; Ginoux P.; Kinne S.; Torres O.; Holben B. N.; Duncan B. N.; Martin R. V.; Logan J. A.; Higurashi A.; Nakajima T. Tropospheric aerosol optical thickness from the GOCART model and comparisons with satellite and Sun photometer measurements. J. Atmos. Sci. 2002, 59 (3), 461–483. 10.1175/1520-0469(2002)059<0461:TAOTFT>2.0.CO;2.

[ref62] Pai S. J.; Heald C. L.; Pierce J. R.; Farina S. C.; Marais E. A.; Jimenez J. L.; Campuzano-Jost P.; Nault B. A.; Middlebrook A. M.; Coe H.; Shilling J. E.; Bahreini R.; Dingle J. H.; Vu K. An evaluation of global organic aerosol schemes using airborne observations. Atmos. Chem. Phys. 2020, 20 (5), 2637–2665. 10.5194/acp-20-2637-2020.

[ref63] Hayes P. L.; Carlton A. G.; Baker K. R.; Ahmadov R.; Washenfelder R. A.; Alvarez S.; Rappenglück B.; Gilman J. B.; Kuster W. C.; de Gouw J. A.; Zotter P.; Prévôt A. S. H.; Szidat S.; Kleindienst T. E.; Offenberg J. H.; Ma P. K.; Jimenez J. L. Modeling the formation and aging of secondary organic aerosols in Los Angeles during CalNex 2010. Atmos. Chem. Phys. 2015, 15 (10), 5773–5801. 10.5194/acp-15-5773-2015.

[ref64] Ford B.; Heald C. L. Aerosol loading in the Southeastern United States: reconciling surface and satellite observations. Atmos. Chem. Phys. 2013, 13 (18), 9269–9283. 10.5194/acp-13-9269-2013.

[ref65] Kim P. S.; Jacob D. J.; Fisher J. A.; Travis K.; Yu K.; Zhu L.; Yantosca R. M.; Sulprizio M. P.; Jimenez J. L.; Campuzano-Jost P.; Froyd K. D.; Liao J.; Hair J. W.; Fenn M. A.; Butler C. F.; Wagner N. L.; Gordon T. D.; Welti A.; Wennberg P. O.; Crounse J. D.; St Clair J. M.; Teng A. P.; Millet D. B.; Schwarz J. P.; Markovic M. Z.; Perring A. E. Sources, seasonality, and trends of southeast US aerosol: an integrated analysis of surface, aircraft, and satellite observations with the GEOS-Chem. chemical transport model. Atmos. Chem. Phys. 2015, 15 (18), 10411–10433. 10.5194/acp-15-10411-2015.

[ref66] McClenny W. A.; Williams E. J.; Cohen R. C.; Stutz J. Preparing to measure the effects of the NO_x_ SIP call-methods for ambient air monitoring of NO, NO_2_, NO_y_, and individual NO_z_ species. J. Air Waste Manage 2002, 52 (5), 542–562. 10.1080/10473289.2002.10470801. 12022694

[ref67] Reed C.; Evans M. J.; Di Carlo P.; Lee J. D.; Carpenter L. J. Interferences in photolytic NO2 measurements: explanation for an apparent missing oxidant?. Atmos. Chem. Phys. 2016, 16 (7), 4707–4724. 10.5194/acp-16-4707-2016.

[ref68] O’Dell K.; Ford B.; Fischer E. V.; Pierce J. R. Contribution of Wildland-Fire Smoke to US PM2.5 and Its Influence on Recent Trends. Environ. Sci. Technol. 2019, 53 (4), 1797–1804. 10.1021/acs.est.8b05430. 30681842

[ref69] Philip S.; Martin R. V.; Pierce J. R.; Jimenez J. L.; Zhang Q.; Canagaratna M. R.; Spracklen D. V.; Nowlan C. R.; Lamsal L. N.; Cooper M. J.; Krotkov N. A. Spatially and seasonally resolved estimate of the ratio of organic mass to organic carbon. Atmos. Environ. 2014, 87, 34–40. 10.1016/j.atmosenv.2013.11.065.

[ref70] Hudman R. C.; Murray L. T.; Jacob D. J.; Millet D. B.; Turquety S.; Wu S.; Blake D. R.; Goldstein A. H.; Holloway J.; Sachse G. W. Biogenic versus anthropogenic sources of CO in the United States. Geophys. Res. Lett. 2008, 35( (4), ). 10.1029/2007GL032393.

[ref71] Miller S. M.; Matross D. M.; Andrews A. E.; Millet D. B.; Longo M.; Gottlieb E. W.; Hirsch A. I.; Gerbig C.; Lin J. C.; Daube B. C.; Hudman R. C.; Dias P. L. S.; Chow V. Y.; Wofsy S. C. Sources of carbon monoxide and formaldehyde in North America determined from high-resolution atmospheric data. Atmos. Chem. Phys. 2008, 8 (24), 7673–7696. 10.5194/acp-8-7673-2008.

[ref72] Dallmann T. R.; Harley R. A. Evaluation of mobile source emission trends in the United States. J. Geophys. Res.: Atmos. 2010, 115. 10.1029/2010JD013862.

[ref73] Dallmann T. R.; Kirchstetter T. W.; DeMartini S. J.; Harley R. A. Quantifying On-Road Emissions from Gasoline-Powered Motor Vehicles: Accounting for the Presence of Medium- and Heavy-Duty Diesel Trucks. Environ. Sci. Technol. 2013, 47 (23), 13873–13881. 10.1021/es402875u. 24215572

[ref74] Cao H. S.; Henze D. K.; Cady-Pereira K.; McDonald B. C.; Harkins C.; Sun K.; Bowman K. W.; Fu T. M.; Nawaz M. O. COVID-19 Lockdowns Afford the First Satellite-Based Confirmation That Vehicles Are an Under-recognized Source of Urban NH_3_ Pollution in Los Angeles. Environ. Sci. Tech Lett. 2022, 9 (1), 3–9. 10.1021/acs.estlett.1c00730.

[ref75] Buonocore J. J.; Salimifard P.; Michanowicz D. R.; Allen J. G. A decade of the US energy mix transitioning away from coal: historical reconstruction of the reductions in the public health burden of energy. Environ. Res. Lett. 2021, 16 (5), 054030 10.1088/1748-9326/abe74c.

[ref76] Gentner D. R.; Isaacman G.; Worton D. R.; Chan A. W. H.; Dallmann T. R.; Davis L.; Liu S.; Day D. A.; Russell L. M.; Wilson K. R.; Weber R.; Guha A.; Harley R. A.; Goldstein A. H. Elucidating secondary organic aerosol from diesel and gasoline vehicles through detailed characterization of organic carbon emissions. Proc. Natl. Acad. Sci. U.S.A. 2012, 109 (45), 18318–18323. 10.1073/pnas.1212272109. 23091031 PMC3494959

[ref86] Heald C. L.; Collett J. L.; Lee T.; Benedict K. B.; Schwandner F. M.; Li Y.; Clarisse L.; Hurtmans D. R.; Van Damme M.; Clerbaux C.; Coheur P. F.; Philip S.; Martin R. V.; Pye H. O. T. Atmospheric ammonia and particulate inorganic nitrogen over the United States. Atmos. Chem. Phys. 2012, 12 (21), 10295–10312. 10.5194/acp-12-10295-2012.

[ref87] Luo G.; Yu F. Q.; Moch J. M. Further improvement of wet process treatments in GEOS-Chem. v12.6.0: impact on global distributions of aerosols and aerosol precursors. Geosci. Model Dev. 2020, 13 (6), 2879–2903. 10.5194/gmd-13-2879-2020.

[ref88] Miao R. Q.; Chen Q.; Zheng Y.; Cheng X.; Sun Y. L.; Palmer P. I.; Shrivastava M.; Guo J. P.; Zhang Q.; Liu Y. H.; Tan Z. F.; Ma X. F.; Chen S. Y.; Zeng L. M.; Lu K. D.; Zhang Y. H. Model bias in simulating major chemical components of PM2.5 in China. Atmos. Chem. Phys. 2020, 20 (20), 12265–12284. 10.5194/acp-20-12265-2020.

[ref89] Liggio J.; Li S. M.; Vlasenko A.; Stroud C.; Makar P. Depression of Ammonia Uptake to Sulfuric Acid Aerosols by Competing Uptake of Ambient Organic Gases. Environ. Sci. Technol. 2011, 45 (7), 2790–2796. 10.1021/es103801g. 21405082

[ref90] Silvern R. F.; Jacob D. J.; Kim P. S.; Marais E. A.; Turner J. R.; Campuzano-Jost P.; Jimenez J. L. Inconsistency of ammonium-sulfate aerosol ratios with thermodynamic models in the eastern US: a possible role of organic aerosol. Atmos. Chem. Phys. 2017, 17 (8), 5107–5118. 10.5194/acp-17-5107-2017.

[ref92] Peeters J.; Müller J. F. HO_*x*_ radical regeneration in isoprene oxidation via peroxy radical isomerisations. II: experimental evidence and global impact. Phys. Chem. Chem. Phys. 2010, 12 (42), 14227–14235. 10.1039/c0cp00811g. 20882226

[ref93] Crounse J. D.; Paulot F.; Kjaergaard H. G.; Wennberg P. O. Peroxy radical isomerization in the oxidation of isoprene. Phys. Chem. Chem. Phys. 2011, 13 (30), 13607–13613. 10.1039/c1cp21330j. 21701740

[ref94] Chan Miller C.; Jacob D. J.; Marais E. A.; Yu K. R.; Travis K. R.; Kim P. S.; Fisher J. A.; Zhu L.; Wolfe G. M.; Hanisco T. F.; Keutsch F. N.; Kaiser J.; Min K. E.; Brown S. S.; Washenfelder R. A.; González Abad G.; Chance K. Glyoxal yield from isoprene oxidation and relation to formaldehyde: chemical mechanism, constraints from SENEX aircraft observations, and interpretation of OMI satellite data. Atmos. Chem. Phys. 2017, 17 (14), 8725–8738. 10.5194/acp-17-8725-2017.

[ref95] Pye H. O. T.; D’Ambro E. L.; Lee B.; Schobesberger S.; Takeuchi M.; Zhao Y.; Lopez-Hilfiker F.; Liu J. M.; Shilling J. E.; Xing J.; Mathur R.; Middlebrook A. M.; Liao J.; Welti A.; Graus M.; Warneke C.; de Gouw J. A.; Holloway J. S.; Ryerson T. B.; Pollack I. B.; Thornton J. A. Anthropogenic enhancements to production of highly oxygenated molecules from autoxidation. Proc. Natl. Acad. Sci. U.S.A. 2019, 116 (14), 6641–6646. 10.1073/pnas.1810774116. 30886090 PMC6452672

[ref96] Chameides W. L.; Lindsay R. W.; Richardson J.; Kiang C. S. The Role of Biogenic Hydrocarbons in Urban Photochemical Smog - Atlanta as a Case-Study. Science 1988, 241 (4872), 1473–1475. 10.1126/science.3420404. 3420404

[ref97] Hettiyadura A. P. S.; Al-Naiema I. M.; Hughes D. D.; Fang T.; Stone E. A. Organosulfates in Atlanta, Georgia: anthropogenic influences on biogenic secondary organic aerosol formation. Atmos. Chem. Phys. 2019, 19 (5), 3191–3206. 10.5194/acp-19-3191-2019.

[ref98] Jung J.; Choi Y.; Mousavinezhad S.; Kang D. W.; Park J.; Pouyaei A.; Ghahremanloo M.; Momeni M.; Kim H. Changes in the ozone chemical regime over the contiguous United States inferred by the inversion of NO_*x*_ and VOC emissions using satellite observation. Atmos. Res. 2022, 270, 106076 10.1016/j.atmosres.2022.106076. PMC8972085 35370333

[ref99] Woody M. C.; Baker K. R.; Hayes P. L.; Jimenez J. L.; Koo B.; Pye H. O. T. Understanding sources of organic aerosol during CalNex-2010 using the CMAQ-VBS. Atmos. Chem. Phys. 2016, 16 (6), 4081–4100. 10.5194/acp-16-4081-2016.

[ref100] Hodzic A.; Jimenez J. L. Modeling anthropogenically controlled secondary organic aerosols in a megacity: a simplified framework for global and climate models. Geosci. Model Dev. 2011, 4 (4), 901–917. 10.5194/gmd-4-901-2011.

[ref101] He M.; Ditto J. C.; Gardner L.; Machesky J.; Hass-Mitchell T. N.; Chen C.; Khare P.; Sahin B.; Fortner J. D.; Plata D. L.; Drollette B. D.; Hayden K. L.; Wentzell J. J. B.; Mittermeier R. L.; Leithead A.; Lee P.; Darlington A.; Wren S. N.; Zhang J.; Wolde M.; Moussa S. G.; Li S.-M.; Liggio J.; Gentner D. R. Total organic carbon measurements reveal major gaps in petrochemical emissions reporting. Science 2024, 383 (6681), 426–432. 10.1126/science.adj6233. 38271520

[ref102] Pinder R. W.; Adams P. J.; Pandis S. N. Ammonia emission controls as a cost-effective strategy for reducing atmospheric particulate matter in the eastern United States. Environ. Sci. Technol. 2007, 41 (2), 380–386. 10.1021/es060379a. 17310695

[ref103] Vayenas D. V.; Takahama S.; Davidson C. I.; Pandis S. N. Simulation of the thermodynamics and removal processes in the sulfate-ammonia-nitric acid system during winter:: Implications for PM_2.5_ control strategies. J. Geophys. Res.: Atmos. 2005, 110( (D7), ). 10.1029/2004JD005038.

[ref104] Zhu L.; Jacob D. J.; Mickley L. J.; Marais E. A.; Cohan D. S.; Yoshida Y.; Duncan B. N.; González Abad G.; Chance K. V. Anthropogenic emissions of highly reactive volatile organic compounds in eastern Texas inferred from oversampling of satellite (OMI) measurements of HCHO columns. Environ. Res. Lett. 2014, 9 (11), 114004 10.1088/1748-9326/9/11/114004.

[ref105] Kelly J. M.; Marais E. A.; Lu G.; Obszynska J.; Mace M.; White J.; Leigh R. J. Diagnosing domestic and transboundary sources of fine particulate matter (PM2.5) in UK cities using GEOS-Chem. City Environ. Interac. 2023, 18, 100100 10.1016/j.cacint.2023.100100.

[ref106] Goodkind A. L.; Tessum C. W.; Coggins J. S.; Hill J. D.; Marshall J. D. Fine-scale damage estimates of particulate matter air pollution reveal opportunities for location-specific mitigation of emissions. Proc. Natl. Acad. Sci. U.S.A. 2019, 116 (18), 8775–8780. 10.1073/pnas.1816102116. 30962364 PMC6500143

[ref107] Anenberg S. C.; Mohegh A.; Goldberg D. L.; Kerr G. H.; Brauer M.; Burkart K.; Hystad P.; Larkin A.; Wozniak S.; Lamsal L. Long-term trends in urban NO_2_ concentrations and associated paediatric asthma incidence: estimates from global datasets. Lancet Planet Health 2022, 6 (1), E49–E58. 10.1016/S2542-5196(21)00255-2. 34998460

[ref108] Marais E. A.; Kelly J. M.; Vohra K.; Li Y.; Lu G.; Hina N.; Rowe E. C. Impact of Legislated and Best Available Emission Control Measures on UK Particulate Matter Pollution, Premature Mortality, and Nitrogen-Sensitive Habitats. GeoHealth 2023, 7 (10), e2023GH000910 10.1029/2023GH000910. PMC10599219 37885915

[ref109] Loh M. M.; Levy J. I.; Spengler J. D.; Houseman E. A.; Bennett D. H. Ranking cancer risks of organic hazardous air pollutants in the United States. Environ. Health Perspect. 2007, 115 (8), 1160–1168. 10.1289/ehp.9884. 17687442 PMC1940102

[ref110] Murray C. J. L.; Aravkin A. Y.; Zheng P.; Abbafati C.; Abbas K. M.; Abbasi-Kangevari M.; Abd-Allah F.; Abdelalim A.; Abdollahi M.; Abdollahpour L.; et al. Global burden of 87 risk factors in 204 countries and territories, 1990–2019: a systematic analysis for the Global Burden of Disease Study 2019. The Lancet 2020, 396 (10258), 1223–1249. 10.1016/S0140-6736(20)30752-2. PMC7566194 33069327

[ref111] Malashock D. A.; DeLang M. N.; Becker J. S.; Serre M. L.; West J. J.; Chang K. L.; Cooper O. R.; Anenberg S. C. Estimates of ozone concentrations and attributable mortality in urban, peri-urban and rural areas worldwide in 2019. Environ. Res. Lett. 2022, 17 (5), 054023 10.1088/1748-9326/ac66f3. 36495890

[ref112] Hao H.; Wang Y. F.; Zhu Q.; Zhang H. S.; Rosenberg A.; Schwartz J.; Amini H.; van Donkelaar A.; Martin R.; Liu P. F.; Weber R.; Russel A.; Yitshak-sade M.; Chang H.; Shi L. H. National Cohort Study of Long-Term Exposure to PM_2.5_ Components and Mortality in Medicare American Older Adults. Environ. Sci. Technol. 2023, 57 (17), 6835–6843. 10.1021/acs.est.2c07064. 37074132 PMC10157884

[ref113] Wang Y. F.; Xiao S. Y.; Zhang Y. H.; Chang H.; Martin R. V.; Van Donkelaar A.; Gaskins A.; Liu Y.; Liu P. F.; Shi L. H. Long-term exposure to PM_2.5_ major components and mortality in the southeastern United States. Environ. Int. 2022, 158, 106969 10.1016/j.envint.2021.106969. 34741960 PMC9190768

[ref114] McFiggans G.; Mentel T. F.; Wildt J.; Pullinen I.; Kang S.; Kleist E.; Schmitt S.; Springer M.; Tillmann R.; Wu C.; Zhao D. F.; Hallquist M.; Faxon C.; Le Breton M.; Hallquist Å. M.; Simpson D.; Bergström R.; Jenkin M. E.; Ehn M.; Thornton J. A.; Alfarra M. R.; Bannan T. J.; Percival C. J.; Priestley M.; Topping D.; Kiendler-Scharr A. Secondary organic aerosol reduced by mixture of atmospheric vapours. Nature 2019, 565 (7741), 587–593. 10.1038/s41586-018-0871-y. 30700872

[ref115] Sharvini S. R.; Noor Z. Z.; Chong C. S.; Stringer L. C.; Yusuf R. O. Energy consumption trends and their linkages with renewable energy policies in East and Southeast Asian countries: Challenges and opportunities. Sustainable Environ. Res. 2018, 28, 257–266. 10.1016/j.serj.2018.08.006.

[ref116] Marais E. A.; Silvern R. F.; Vodonos A.; Dupin E.; Bockarie A. S.; Mickley L. J.; Schwartz J. Air quality and health impact of future fossil fuel use for electricity generation and transport in Africa. Environ. Sci. Technol. 2019, 53, 13524–13534. 10.1021/acs.est.9b04958. 31647871

[ref117] SLOCAT. Africa – SLOCAT Transport and Climate Change Global Status Report. 2021. https://tcc-gsr.com/global-overview/africa/. accessed 10 July 2024.

