# Supplementary material for: Influence of Oil and Gas End-Use on Summertime Particulate Matter and Ozone Pollution in the Eastern US
Source: Environ Sci Technol. 2024 Oct 17;58(44):19736–47. doi: 10.1021/acs.est.4c10032 (PMC11542890; doi:10.1021/acs.est.4c10032)
Supplement: Supplementary file 1 — es4c10032_si_001.pdf [file es4c10032_si_001.pdf]

Supporting Information for

Influence of Oil and Gas End-Use on Summertime Particulate Matter and Ozone Pollution in the Eastern US

Karn Vohra[1*], Eloise A. Marais[1*], Ploy Achakulwisut[2], Gongda Lu[1], Jamie M. Kelly[1a], Colin Harkins[3,4], and Brian McDonald[4]

[1] Department of Geography, University College London, London WC1E 6BT, UK.

[2] Stockholm Environment Institute US, Seattle 98101, WA, USA.

[3] Cooperative Institute for Research in Environmental Sciences, University of Colorado Boulder, Boulder 80309, CO, USA.

[4] NOAA Chemical Sciences Laboratory, Boulder 80305, CO, USA.

[a] Now at: Centre for Research and Clean Air, Helsinki 100810, Finland

Corresponding authors: Eloise A. Marais (e.marais@ucl.ac.uk) and Karn Vohra (k.vohra@bham.ac.uk)

Summary: 12 pages, 2 figures, 1 table.

Pages S2-S13 include

Supplementary Text S1

Figures S1-S2

Supplementary Table S1

References

Supplement Text S1. GEOS-Chem Consistency with Observations

Figure S1 compares simulated and observed summertime mean surface concentrations of gas-phase compounds (NO_2^* and MDA8 O_3) across eastern US in 2017. According to the observations, NO_2 in urban areas in summer 2017 is ~13-15 ppb in densely populated cities like New York City and Philadelphia, 6-8 ppb along the I-95 interstate highway extending along the northeast coast, and <2 ppb in remote rural areas. The model moderately reproduces the spatial variability in NO_2^* ($R = 0.61$) and is on average very consistent with the observations (model normalized mean bias or NMB of -0.1%). At individual sites, though, the model typically differs from the observations by ~4-6 ppb, as the resolution of the model (~25 km) is too coarse to reproduce steep urban-to-rural NO_2 gradients[1-3].

Observed MDA8 O_3 exceeds 30 ppb across more than 90% of the monitoring sites in eastern US and peaks at 50-53 ppb in Baltimore, St. Louis, and Chicago. NO_x titration of O_3 is most pronounced at night, whereas O_3 peaks during the day, so the titration effect is dampened in MDA8 O_3. For model grid cells sampling Chicago, for example, 24-hour mean O_3 is ~5-8 ppb less than it is for surrounding grid cells, whereas there is no significant spatial difference in MDA8 O_3. The model reproduces the observed spatial distribution of MDA8 O_3 ($R = 0.81$), but overestimates background MDA8 O_3 by ~10 ppb, causing a model NMB of ~18%. A 10 ppb bias in modelled O_3 is well documented for the US[4-6] and other parts of the world[7,8]. Part (~3 ppb) of the model bias is due to the higher altitude of the midpoint of the lowest model layer (65 m) compared to the sampling inlets of the network instruments (~3-5 m above ground level). This altitude difference causes an underestimate in modelled O_3 dry deposition[5], a major surface O_3 loss process[9].

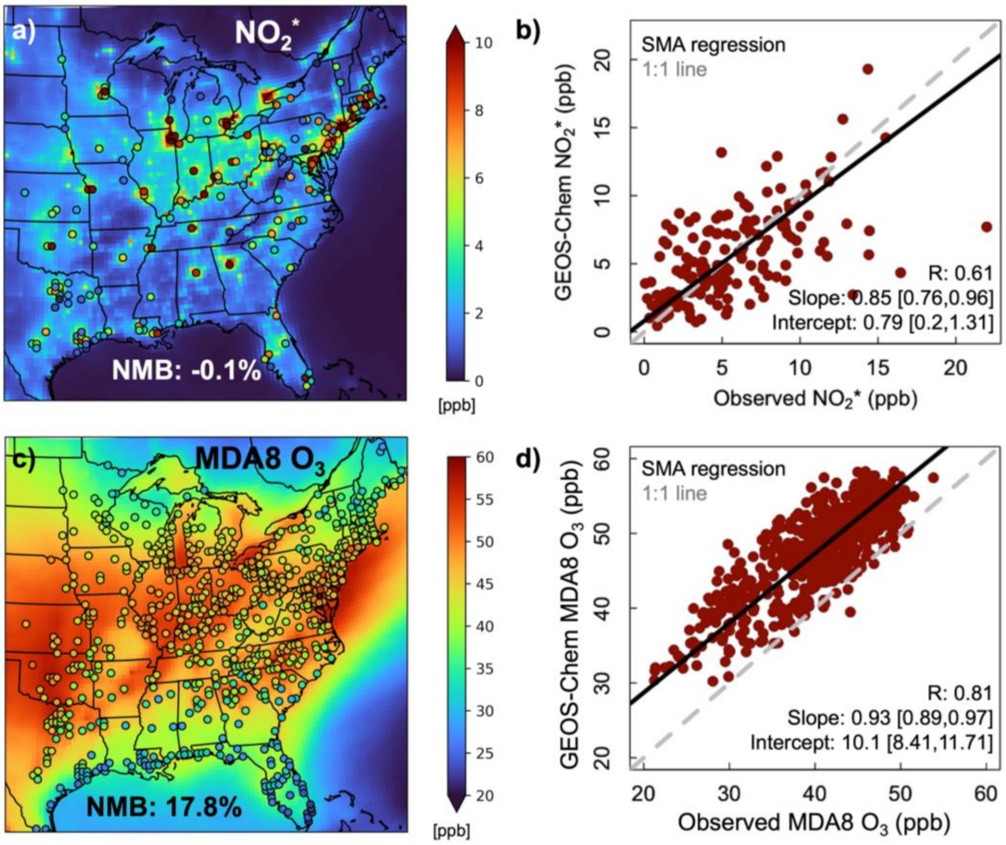

Figure S1. Assessment of GEOS-Chem eastern US summer 2017 surface NO_2^* and MDA8 O_3. Maps compare simulated (background) and observed (circles) June-August mean NO_2^* (a) and MDA8 O_3 (c). Values inset are the model normalized mean bias (NMB) for coincident grid squares and observations. Scatter plots compare coincident modelled and observed NO_2^* (b) and MDA8 O_3 (d). Lines are the standard major axis (SMA) regression (black solid) and 1:1 agreement (grey dashed). Values inset are Pearson's correlation coefficients (R) and SMA regression statistics. Relative errors on the slopes and intercepts are 95 % confidence intervals (CI).

Figure S2 compares simulated and observed summer 2017 mean surface concentrations of particle-phase pollutants ($PM_{2.5}$ and OC) across eastern US. Observed $PM_{2.5}$ ranges from ~8-10 μg m^{-3} across most of eastern US to a regional hotspot of 13-14 μg m^{-3} covering Illinois, Indiana, and Ohio. There is moderate model agreement with the spatial distribution of observed $PM_{2.5}$ (R = 0.51), but an overestimate of ~41% or 2.8 μg m^{-3} in background $PM_{2.5}$ (intercept of 2.8 μg m^{-3} in Figure S2(b)). We examine individual $PM_{2.5}$ components OC, pNO_3, pNH_4, and pSO_4 (not shown) to diagnose the drivers of this large model bias. For all 4 components, the model is spatially consistent with the observations (R of 0.63 for OC, 0.68 for pNO_3, 0.76 for pNH_4, and 0.60 for pSO_4). For OC, the regression statistics are sensitive to the choice of linear

fit: slope of 1.66 and intercept of -1.22 µg m^{-3} using SMA regression and slope of 0.98 and intercept of 0.18 µg m^{-3} using Theil-Sen regression. This could be because of limited variability in simulated and observed OC and influence of the population of points with modelled OC > 3.5 µg m^{-3} in New York and Philadelphia. The model OC NMB is only 9.9%, equivalent to 0.2-0.3 µg C m^{-3} or 0.4-0.6 µg m^{-3} OA using an OA/OC ratio of 2.1.

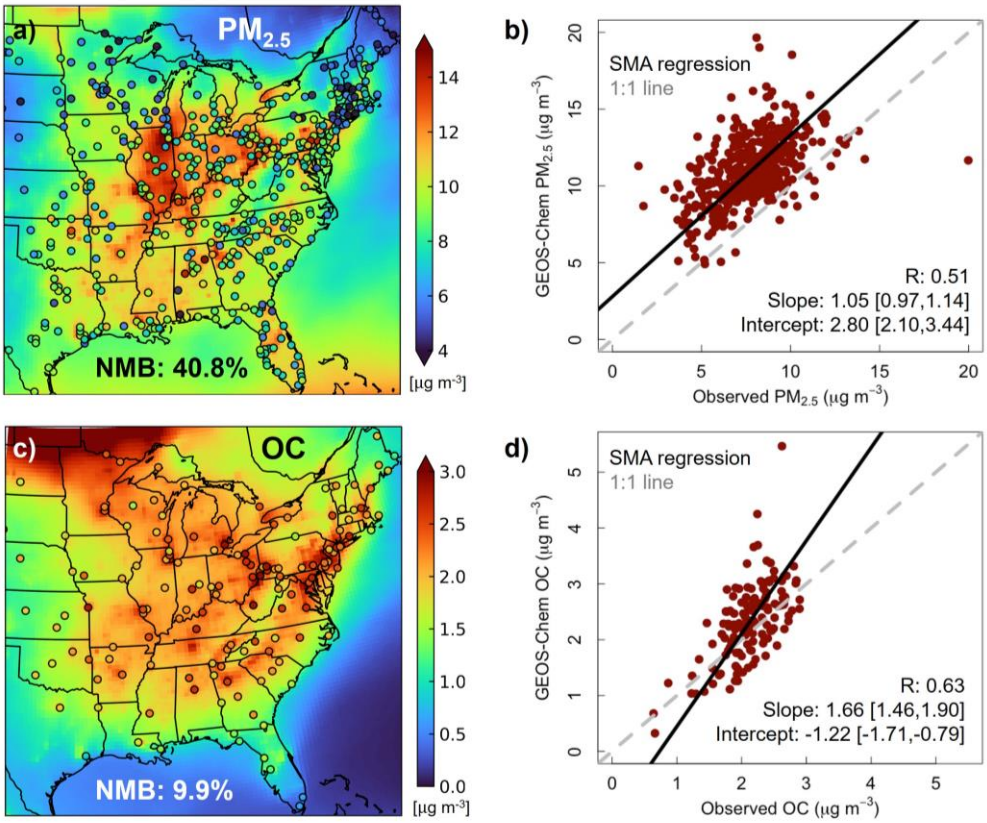

Figure S2. Assessment of GEOS-Chem eastern US summer 2017 surface $PM_{2.5}$ and OC. Maps compare simulated (background) and observed (circles) June-August mean $PM_{2.5}$ (a) and OC (c). OC from modelled SOA is calculated using OA/OC = 2.1. Values inset are the model NMB for coincident grid squares and observations. Scatter plots compare coincident modelled and observed $PM_{2.5}$ (b) and OC (d). Lines are SMA regression (black solid) and 1:1 agreement (grey dashed). Values inset are Pearson's correlation coefficients (R) and SMA regression statistics. Relative errors on the slopes and intercepts are the 95 % CI.

Network observations of pSO_4 in the eastern US typically range from 0.8 to 1.4 µg m^{-3}. The model pSO_4 NMB is only 12% or ~0.1 µg m^{-3}, due to a small model overestimate in variance (slope = 1.04) and background concentrations (intercept = 0.09 µg m^{-3}).

GEOS-Chem overestimates each component by almost a factor of 3 (NMBs of 181% for pNO_3 and 195% for pNH_4, Section 3.2). Even with the large model bias in pNO_3 and pNH_4, these each make a smaller contribution (≤5%) to $PM_{2.5}$ than OA (~35%) or pSO_4 (~10%). A 3-fold model overestimate in pNO_3 and pNH_4 amounting to ~0.3 μg m^{-3} each, along with the small model bias in OA (~0.5 μg m^{-3} for OA/OC = 2.1) and pSO_4 (~0.1 μg m^{-3}), and the associated aerosol water for all these components (Equation (1)), totals ~1.2 μg m^{-3}; less than half the 2.8 μg m^{-3} model overestimate in eastern US $PM_{2.5}$.

Dust is a dominant component of modelled $PM_{2.5}$ (39%). Anthropogenic dust emissions are uncertain[10] and our attribution of "Other $PM_{2.5}$" in the NEI inventory to dust (Section 2.1) due to absence of information about the specific composition of this class of $PM_{2.5}$ may be erroneous. This "Other $PM_{2.5}$" is mostly (98%) from non-end-use activities. In the absence of direct measurements of dust, we assess the effect of excluding dust in Equation (1) on modelled $PM_{2.5}$ compared to the observations. The slope is relatively unaffected, decreasing from 1.05 to 0.96 with a 95% confidence interval (CI) of 0.88-1.05 that overlaps with the CI of the slope in Figure 2(b). The intercept declines significantly from +2.8 μg m^{-3} to -0.64 μg m^{-3} [95% CI: -1.36 μg m^{-3}, 0.02 μg m^{-3}]. This suggests that model bias in dust likely accounts for most of the remaining (~1.6 μg m^{-3}) overestimate in modelled $PM_{2.5}$.

Supplementary Table S1. List of US Environmental Protection Agency Source Classification Codes (SCC) classified as oil and gas end-use activity.

SCC	Sector	Description
10100401	Fuel Comb - Electric Generation - Oil	Residual Oil - Grade 6: Boiler, Normal Firing
10100404	Fuel Comb - Electric Generation - Oil	Residual Oil - Grade 6: Boiler, Tangential-fired
10100501	Fuel Comb - Electric Generation - Oil	Distillate Oil - Grades 1 and 2: Boiler
10100504	Fuel Comb - Electric Generation - Oil	Distillate Oil - Grade 4: Boiler, Normal Firing
10100505	Fuel Comb - Electric Generation - Oil	Distillate Oil - Grade 4: Boiler, Tangential-fired
10100601	Fuel Comb - Electric Generation - Natural Gas	Boiler, >= 100 Million BTU/hr
10100602	Fuel Comb - Electric Generation - Natural Gas	Boiler < 100 Million BTU, except tangential
10100604	Fuel Comb - Electric Generation - Natural Gas	Boiler, Tangential-fired
10100701	Fuel Comb - Electric Generation - Other	Boiler, >= 100 Million BTU/hr
10100702	Fuel Comb - Electric Generation - Other	Boiler < 100 Million Btu/hr
10100703	Fuel Comb - Electric Generation - Other	Petroleum Refinery Gas: Boiler
10100711	Fuel Comb - Electric Generation - Other	Landfill Gas
10100712	Fuel Comb - Electric Generation - Other	Digester Gas
10101001	Fuel Comb - Electric Generation - Other	Butane
10101002	Fuel Comb - Electric Generation - Other	Propane
10200401	Fuel Comb - Industrial Boilers, ICEs - Oil	Grade 6 oil
10200402	Fuel Comb - Industrial Boilers, ICEs - Oil	10-100 Million BTU/hr
10200403	Fuel Comb - Industrial Boilers, ICEs - Oil	< 10 Million BTU/hr
10200404	Fuel Comb - Industrial Boilers, ICEs - Oil	Grade 5 Oil
10200405	Fuel Comb - Industrial Boilers, ICEs - Oil	Cogeneration
10200406	Fuel Comb - Industrial Boilers, ICEs - Oil	Boiler > 100 Million BTU/hr
10200501	Fuel Comb - Industrial Boilers, ICEs - Oil	Distillate Oil - Grades 1 and 2: Boiler
10200502	Fuel Comb - Industrial Boilers, ICEs - Oil	10-100 Million BTU/hr
10200503	Fuel Comb - Industrial Boilers, ICEs - Oil	< 10 Million BTU/hr
10200504	Fuel Comb - Industrial Boilers, ICEs - Oil	Grade 4 Oil
10200505	Fuel Comb - Industrial Boilers, ICEs - Oil	Cogeneration
10200506	Fuel Comb - Industrial Boilers, ICEs - Oil	Boiler > 100 Million BTU/hr
10200601	Fuel Comb - Industrial Boilers, ICEs - Natural Gas	> 100 Million BTU/hr
10200602	Fuel Comb - Industrial Boilers, ICEs - Natural Gas	10-100 Million BTU/hr
10200603	Fuel Comb - Industrial Boilers, ICEs - Natural Gas	< 10 Million BTU/hr
10200604	Fuel Comb - Industrial Boilers, ICEs - Natural Gas	Cogeneration
10200701	Fuel Comb - Industrial Boilers, ICEs - Other	Petroleum Refinery Gas
10200704	Fuel Comb - Industrial Boilers, ICEs - Other	Blast Furnace Gas
10200707	Fuel Comb - Industrial Boilers, ICEs - Other	Coke Oven Gas
10200710	Fuel Comb - Industrial Boilers, ICEs - Other	Cogeneration
10200711	Fuel Comb - Industrial Boilers, ICEs - Other	Landfill Gas
10200799	Fuel Comb - Industrial Boilers, ICEs - Other	Other: Specify in Comments
10201001	Fuel Comb - Industrial Boilers, ICEs - Other	Butane
10201002	Fuel Comb - Industrial Boilers, ICEs - Other	Propane

**SCC**	**Sector**	**Description**
10201003	Fuel Comb - Industrial Boilers, ICEs - Other	Butane/Propane Mixture: Specify Percent Butane in Comments
10300401	Fuel Comb - Comm/Institutional - Oil	Residual Oil - Grade 6: Boiler
10300402	Fuel Comb - Comm/Institutional - Oil	10-100 Million BTU/hr
10300403	Fuel Comb - Comm/Institutional - Oil	< 10 Million BTU/hr
10300404	Fuel Comb - Comm/Institutional - Oil	Grade 5 Oil
10300405	Fuel Comb - Comm/Institutional - Oil	Boiler > 100 Million BTU/hr
10300501	Fuel Comb - Comm/Institutional - Oil	Distillate Oil - Grades 1 and 2: Boiler
10300502	Fuel Comb - Comm/Institutional - Oil	10-100 Million BTU/hr
10300503	Fuel Comb - Comm/Institutional - Oil	< 10 Million BTU/hr
10300504	Fuel Comb - Comm/Institutional - Oil	Grade 4 Oil
10300505	Fuel Comb - Comm/Institutional - Oil	Boiler > 100 Million BTU/hr
10300601	Fuel Comb - Comm/Institutional - Natural Gas	> 100 Million BTU/hr
10300602	Fuel Comb - Comm/Institutional - Natural Gas	10-100 Million BTU/hr
10300603	Fuel Comb - Comm/Institutional - Natural Gas	< 10 Million BTU/hr
10300701	Fuel Comb - Comm/Institutional - Other	POTW Digester Gas-fired Boiler
10300799	Fuel Comb - Comm/Institutional - Other	Other Not Classified
10301001	Fuel Comb - Comm/Institutional - Other	Butane
10301002	Fuel Comb - Comm/Institutional - Other	Propane
10301003	Fuel Comb - Comm/Institutional - Other	Butane/Propane Mixture: Specify Percent Butane in Comments
10500105	Fuel Comb - Industrial Boilers, ICEs - Oil	Distillate Oil
10500106	Fuel Comb - Industrial Boilers, ICEs - Natural Gas	Natural Gas
10500110	Fuel Comb - Industrial Boilers, ICEs - Other	Liquified Petroleum Gas (LPG)
10500113	Fuel Comb - Industrial Boilers, ICEs - Other	Waste Oil: Air Atomized Burner
10500114	Fuel Comb - Industrial Boilers, ICEs - Other	Waste Oil: Vaporizing Burner
10500205	Fuel Comb - Comm/Institutional - Oil	Distillate Oil
10500206	Fuel Comb - Comm/Institutional - Natural Gas	Natural Gas
10500210	Fuel Comb - Comm/Institutional - Other	Liquified Petroleum Gas (LPG)
10500213	Fuel Comb - Comm/Institutional - Other	Waste Oil: Air Atomized Burner
10500214	Fuel Comb - Comm/Institutional - Other	Waste Oil: Vaporizing Burner
20100101	Fuel Comb - Electric Generation - Oil	Turbine
20100102	Fuel Comb - Electric Generation - Oil	Reciprocating
20100105	Fuel Comb - Electric Generation - Oil	Reciprocating: Crankcase Blowby
20100106	Fuel Comb - Electric Generation - Oil	Reciprocating: Evaporative Losses (Fuel Storage and Delivery System)
20100107	Fuel Comb - Electric Generation - Oil	Reciprocating: Exhaust
20100108	Fuel Comb - Electric Generation - Oil	Turbine: Evaporative Losses (Fuel Storage and Delivery System)
20100109	Fuel Comb - Electric Generation - Oil	Turbine: Exhaust
20100201	Fuel Comb - Electric Generation - Natural Gas	Turbine
20100202	Fuel Comb - Electric Generation - Natural Gas	Reciprocating
20100205	Fuel Comb - Electric Generation - Natural Gas	Reciprocating: Crankcase Blowby
20100206	Fuel Comb - Electric Generation - Natural Gas	Reciprocating: Evaporative Losses (Fuel Delivery System)

SCC	Sector	Description
20100207	Fuel Comb - Electric Generation - Natural Gas	Reciprocating: Exhaust
20100208	Fuel Comb - Electric Generation - Natural Gas	Turbine: Evaporative Losses (Fuel Delivery System)
20100209	Fuel Comb - Electric Generation - Natural Gas	Turbine: Exhaust
20100702	Fuel Comb - Electric Generation - Other	Reciprocating
20100707	Fuel Comb - Electric Generation - Other	Reciprocating: Exhaust
20100901	Fuel Comb - Electric Generation - Oil	Turbine
20100902	Fuel Comb - Electric Generation - Oil	Reciprocating
20100908	Fuel Comb - Electric Generation - Oil	Turbine: Evaporative Losses (Fuel Storage and Delivery System)
20100909	Fuel Comb - Electric Generation - Oil	Turbine: Exhaust
20200101	Fuel Comb - Industrial Boilers, ICEs - Oil	Turbine
20200102	Fuel Comb - Industrial Boilers, ICEs - Oil	Reciprocating
20200103	Fuel Comb - Industrial Boilers, ICEs - Oil	Turbine: Cogeneration
20200104	Fuel Comb - Industrial Boilers, ICEs - Oil	Reciprocating: Cogeneration
20200105	Fuel Comb - Industrial Boilers, ICEs - Oil	Reciprocating: Crankcase Blowby
20200106	Fuel Comb - Industrial Boilers, ICEs - Oil	Reciprocating: Evaporative Losses (Fuel Storage and Delivery System)
20200107	Fuel Comb - Industrial Boilers, ICEs - Oil	Reciprocating: Exhaust
20200108	Fuel Comb - Industrial Boilers, ICEs - Oil	Turbine: Evaporative Losses (Fuel Storage and Delivery System)
20200109	Fuel Comb - Industrial Boilers, ICEs - Oil	Turbine: Exhaust
20200201	Fuel Comb - Industrial Boilers, ICEs - Natural Gas	Turbine
20200202	Fuel Comb - Industrial Boilers, ICEs - Natural Gas	Reciprocating
20200203	Fuel Comb - Industrial Boilers, ICEs - Natural Gas	Turbine: Cogeneration
20200204	Fuel Comb - Industrial Boilers, ICEs - Natural Gas	Reciprocating: Cogeneration
20200205	Fuel Comb - Industrial Boilers, ICEs - Natural Gas	Reciprocating: Crankcase Blowby
20200206	Fuel Comb - Industrial Boilers, ICEs - Natural Gas	Reciprocating: Evaporative Losses (Fuel Delivery System)
20200207	Fuel Comb - Industrial Boilers, ICEs - Natural Gas	Reciprocating: Exhaust
20200209	Fuel Comb - Industrial Boilers, ICEs - Natural Gas	Turbine: Exhaust
20200251	Fuel Comb - Industrial Boilers, ICEs - Natural Gas	2-cycle Rich Burn
20200252	Fuel Comb - Industrial Boilers, ICEs - Natural Gas	2-cycle Lean Burn
20200253	Fuel Comb - Industrial Boilers, ICEs - Natural Gas	4-cycle Rich Burn
20200254	Fuel Comb - Industrial Boilers, ICEs - Natural Gas	4-cycle Lean Burn
20200255	Fuel Comb - Industrial Boilers, ICEs - Natural Gas	2-cycle Clean Burn
20200256	Fuel Comb - Industrial Boilers, ICEs - Natural Gas	4-cycle Clean Burn
20200401	Fuel Comb - Industrial Boilers, ICEs - Oil	Diesel: Large Bore Engine
20200402	Fuel Comb - Industrial Boilers, ICEs - Oil	Dual Fuel (Oil/Gas): Large Bore Engine
20200403	Fuel Comb - Industrial Boilers, ICEs - Oil	Dual Fuel: Large Bore Engine: Cogeneration
20200406	Fuel Comb - Industrial Boilers, ICEs - Oil	Large Bore Engine: Evaporative Losses (Fuel Storage and Delivery System)
20200407	Fuel Comb - Industrial Boilers, ICEs - Oil	Large Bore Engine: Exhaust
20200501	Fuel Comb - Industrial Boilers, ICEs - Oil	Reciprocating
20200506	Fuel Comb - Industrial Boilers, ICEs - Oil	Reciprocating: Evaporative Losses (Fuel Storage and Delivery System)

SCC	Sector	Description
20200702	Fuel Comb - Industrial Boilers, ICEs - Other	Reciprocating Engine
20200705	Fuel Comb - Industrial Boilers, ICEs - Other	Refinery Gas: Turbine
20200712	Fuel Comb - Industrial Boilers, ICEs - Other	Reciprocating: Exhaust
20200714	Fuel Comb - Industrial Boilers, ICEs - Other	Turbine: Exhaust
20200901	Fuel Comb - Industrial Boilers, ICEs - Oil	Turbine
20200902	Fuel Comb - Industrial Boilers, ICEs - Oil	Reciprocating
20200907	Fuel Comb - Industrial Boilers, ICEs - Oil	Reciprocating: Exhaust
20200909	Fuel Comb - Industrial Boilers, ICEs - Oil	Turbine: Exhaust
20201001	Fuel Comb - Industrial Boilers, ICEs - Other	Propane: Reciprocating
20201002	Fuel Comb - Industrial Boilers, ICEs - Other	Butane: Reciprocating
20201005	Fuel Comb - Industrial Boilers, ICEs - Other	Reciprocating: Crankcase Blowby
20201007	Fuel Comb - Industrial Boilers, ICEs - Other	Reciprocating: Exhaust
20201011	Fuel Comb - Industrial Boilers, ICEs - Other	Turbine
20201012	Fuel Comb - Industrial Boilers, ICEs - Other	Reciprocating Engine
20201013	Fuel Comb - Industrial Boilers, ICEs - Other	Turbine: Cogeneration
20201701	Fuel Comb - Industrial Boilers, ICEs - Other	Turbine
20201702	Fuel Comb - Industrial Boilers, ICEs - Other	Reciprocating Engine
20201706	Fuel Comb - Industrial Boilers, ICEs - Other	Reciprocating: Evaporative Losses (Fuel Storage and Delivery System)
20201707	Fuel Comb - Industrial Boilers, ICEs - Other	Reciprocating: Exhaust
20300101	Fuel Comb - Comm/Institutional - Oil	Reciprocating
20300102	Fuel Comb - Comm/Institutional - Oil	Turbine
20300106	Fuel Comb - Comm/Institutional - Oil	Reciprocating: Evaporative Losses (Fuel Storage and Delivery System)
20300107	Fuel Comb - Comm/Institutional - Oil	Reciprocating: Exhaust
20300108	Fuel Comb - Comm/Institutional - Oil	Turbine: Evaporative Losses (Fuel Storage and Delivery System)
20300109	Fuel Comb - Comm/Institutional - Oil	Turbine: Exhaust
20300201	Fuel Comb - Comm/Institutional - Natural Gas	Reciprocating
20300202	Fuel Comb - Comm/Institutional - Natural Gas	Turbine
20300203	Fuel Comb - Comm/Institutional - Natural Gas	Turbine: Cogeneration
20300204	Fuel Comb - Comm/Institutional - Natural Gas	Reciprocating: Cogeneration
20300205	Fuel Comb - Comm/Institutional - Natural Gas	Reciprocating: Crankcase Blowby
20300206	Fuel Comb - Comm/Institutional - Natural Gas	Reciprocating: Evaporative Losses (Fuel Delivery System)
20300207	Fuel Comb - Comm/Institutional - Natural Gas	Reciprocating: Exhaust
20300209	Fuel Comb - Comm/Institutional - Natural Gas	Turbine: Exhaust
20300301	Fuel Comb - Comm/Institutional - Other	Reciprocating
20300307	Fuel Comb - Comm/Institutional - Other	Reciprocating: Exhaust
20300401	Fuel Comb - Comm/Institutional - Oil	Large Bore Engine
20300901	Fuel Comb - Comm/Institutional - Oil	Turbine: JP-4
20301001	Fuel Comb - Comm/Institutional - Other	Propane: Reciprocating
20301002	Fuel Comb - Comm/Institutional - Other	Butane: Reciprocating
20301007	Fuel Comb - Comm/Institutional - Other	Reciprocating: Exhaust

SCC	Sector	Description
20400101	Fuel Comb - Industrial Boilers, ICEs - Oil	Turbojet
20400102	Fuel Comb - Industrial Boilers, ICEs - Oil	Turboshaft
20400110	Fuel Comb - Industrial Boilers, ICEs - Oil	Jet A Fuel
20400111	Fuel Comb - Industrial Boilers, ICEs - Oil	JP-5 Fuel
20400112	Fuel Comb - Industrial Boilers, ICEs - Oil	JP-4 Fuel
20400199	Fuel Comb - Industrial Boilers, ICEs - Other	Other Not Classified
20400201	Fuel Comb - Industrial Boilers, ICEs - Other	Rocket Motor: Solid Propellant
20400202	Fuel Comb - Industrial Boilers, ICEs - Other	Liquid Propellant
20400299	Fuel Comb - Industrial Boilers, ICEs - Other	Other Not Classified
20400301	Fuel Comb - Industrial Boilers, ICEs - Natural Gas	Natural Gas
20400302	Fuel Comb - Industrial Boilers, ICEs - Oil	Diesel/Kerosene
20400303	Fuel Comb - Industrial Boilers, ICEs - Oil	Distillate Oil
20400305	Fuel Comb - Industrial Boilers, ICEs - Oil	Kerosene/Naphtha
20400399	Fuel Comb - Industrial Boilers, ICEs - Other	Other Not Classified
20400401	Fuel Comb - Industrial Boilers, ICEs - Other	Gasoline
20400402	Fuel Comb - Industrial Boilers, ICEs - Oil	Diesel/Kerosene
20400403	Fuel Comb - Industrial Boilers, ICEs - Oil	Distillate Oil
20400404	Fuel Comb - Industrial Boilers, ICEs - Other	Process Gas
20400406	Fuel Comb - Industrial Boilers, ICEs - Oil	Kerosene/Naphtha (Jet Fuel)
20400407	Fuel Comb - Industrial Boilers, ICEs - Oil	Dual Fuel (Gas/Oil)
20400408	Fuel Comb - Industrial Boilers, ICEs - Oil	Residual Oil/Crude Oil
20400409	Fuel Comb - Industrial Boilers, ICEs - Other	Liquified Petroleum Gas (LPG)
20400499	Fuel Comb - Industrial Boilers, ICEs - Other	Other Not Classified
39000402	Industrial Processes - NEC	Cement Kiln/Dryer
39000403	Industrial Processes - NEC	Lime Kiln
39000499	Industrial Processes - NEC	In process fuel use not elsewhere classified
39000501	Industrial Processes - NEC	Asphalt Dryer
39000502	Industrial Processes - NEC	Cement Kiln/Dryer
39000503	Industrial Processes - NEC	Lime Kiln
39000599	Industrial Processes - NEC	In process fuel use not elsewhere classified
39000602	Industrial Processes - NEC	Cement Kiln/Dryer
39000603	Industrial Processes - NEC	Lime Kiln
39000605	Industrial Processes - NEC	Metal Melting
39000699	Industrial Processes - NEC	In process fuel use not elsewhere classified
39000701	Industrial Processes - NEC	Coke Oven or Blast Furnace
39000702	Industrial Processes - NEC	Coke Oven Gas
39000797	Industrial Processes - NEC	General
39001099	Industrial Processes - NEC	In process fuel use not elsewhere classified
39900501	Industrial Processes - NEC	Distillate Oil
39900601	Industrial Processes - NEC	Natural Gas
39900701	Industrial Processes - NEC	Process Gas

SCC	Sector	Description
39901001	Industrial Processes - NEC	LPG
39990001	Industrial Processes - NEC	Distillate Oil (No. 2): Process Heaters
39990002	Industrial Processes - NEC	Residual Oil: Process Heaters
39990003	Industrial Processes - NEC	Natural Gas: Process Heaters
39990004	Industrial Processes - NEC	Process Gas: Process Heaters
39990013	Industrial Processes - NEC	Natural Gas: Incinerators
39990014	Industrial Processes - NEC	Process Gas: Incinerators
39990021	Industrial Processes - NEC	Distillate Oil (No. 2): Flares
39990023	Industrial Processes - NEC	Natural Gas: Flares
39990024	Industrial Processes - NEC	Process Gas: Flares
2102004000	Fuel Comb - Industrial Boilers, ICEs - Oil	Total: Boilers and IC Engines
2102004001	Fuel Comb - Industrial Boilers, ICEs - Oil	All Boiler Types
2102004002	Fuel Comb - Industrial Boilers, ICEs - Oil	All IC Engine Types
2102005000	Fuel Comb - Industrial Boilers, ICEs - Oil	Total: All Boiler Types
2102006000	Fuel Comb - Industrial Boilers, ICEs - Natural Gas	Total: Boilers and IC Engines
2102007000	Fuel Comb - Industrial Boilers, ICEs - Other	Total: All Boiler Types
2102010000	Fuel Comb - Industrial Boilers, ICEs - Other	Total: All Boiler Types
2102011000	Fuel Comb - Industrial Boilers, ICEs - Oil	Total: All Boiler Types
2103004000	Fuel Comb - Comm/Institutional - Oil	Total: Boilers and IC Engines
2103004001	Fuel Comb - Comm/Institutional - Oil	Boilers
2103004002	Fuel Comb - Comm/Institutional - Oil	IC Engines
2103005000	Fuel Comb - Comm/Institutional - Oil	Total: All Boiler Types
2103006000	Fuel Comb - Comm/Institutional - Natural Gas	Total: Boilers and IC Engines
2103007000	Fuel Comb - Comm/Institutional - Other	Total: All Combustor Types
2103011000	Fuel Comb - Comm/Institutional - Oil	Total: All Combustor Types
2104004000	Fuel Comb - Residential - Oil	Total: All Combustor Types
2104006000	Fuel Comb - Residential - Natural Gas	Total: All Combustor Types
2104007000	Fuel Comb - Residential - Other	Total: All Combustor Types
2104011000	Fuel Comb - Residential - Oil	Total: All Heater Types
2265008005	Mobile - Non-Road Equipment - Gasoline	4-Stroke Airport Ground Support Equipment
2270008005	Mobile - Non-Road Equipment - Diesel	Airport Ground Support Equipment
2275001000	Mobile - Aircraft	Total
2280002101	Mobile - Commercial Marine Vessels	C1C2 Port emissions: Main Engine
2280002102	Mobile - Commercial Marine Vessels	C1C2 Port emissions: Auxiliary Engine
2280002103	Mobile - Commercial Marine Vessels	C3 Port emissions: Main Engine
2280002104	Mobile - Commercial Marine Vessels	C3 Port emissions: Auxiliary Engine
2280002201	Mobile - Commercial Marine Vessels	C1C2 Underway emissions: Main Engine
2280002202	Mobile - Commercial Marine Vessels	C1C2 Underway emissions: Auxiliary Engine
2280002203	Mobile - Commercial Marine Vessels	C3 Underway emissions: Main Engine
2280002204	Mobile - Commercial Marine Vessels	C3 Underway emissions: Auxiliary Engine
2280003103	Mobile - Commercial Marine Vessels	C3 Port emissions: Main Engine

SCC	Sector	Description
2280003104	Mobile - Commercial Marine Vessels	C3 Port emissions: Auxiliary Engine
2280003203	Mobile - Commercial Marine Vessels	C3 Underway emissions: Main Engine
2280003204	Mobile - Commercial Marine Vessels	C3 Underway emissions: Auxiliary Engine
2285002006	Mobile - Locomotives	Line Haul Locomotives: Class I Operations
2285002007	Mobile - Locomotives	Line Haul Locomotives: Class II / III Operations
2285002008	Mobile - Locomotives	Line Haul Locomotives: Passenger Trains (Amtrak)
2285002009	Mobile - Locomotives	Line Haul Locomotives: Commuter Lines
2285002010	Mobile - Locomotives	Yard Locomotives

References

(1) Mohegh, A.; Goldberg, D.; Achakulwisut, P.; Anenberg, S. C. Sensitivity of estimated NO_2-attributable pediatric asthma incidence to grid resolution and urbanicity. *Environ Res Lett* **2021**, *16* (1). DOI: 10.1088/1748-9326/abce25.

(2) Li, C.; Martin, R. V.; Cohen, R. C.; Bindle, L.; Zhang, D. D.; Chatterjee, D.; Weng, H. J.; Lin, J. T. Variable effects of spatial resolution on modeling of nitrogen oxides. *Atmos Chem Phys* **2023**, *23* (5), 3031-3049. DOI: 10.5194/acp-23-3031-2023.

(3) Liu, F.; Ronald, J. V.; Eskes, H.; Ding, J. Y.; Mijling, B. Evaluation of modeling NO_2 concentrations driven by satellite-derived and bottom-up emission inventories using in situ measurements over China. *Atmos Chem Phys* **2018**, *18* (6), 4171-4186. DOI: 10.5194/acp-18-4171-2018.

(4) Travis, K. R.; Jacob, D. J.; Fisher, J. A.; Kim, P. S.; Marais, E. A.; Zhu, L.; Yu, K.; Miller, C. C.; Yantosca, R. M.; Sulprizio, M. P.; et al. Why do models overestimate surface ozone in the Southeast United States? *Atmos Chem Phys* **2016**, *16* (21), 13561-13577. DOI: 10.5194/acp-16-13561-2016.

(5) Travis, K. R.; Jacob, D. J. Systematic bias in evaluating chemical transport models with maximum daily 8 h average (MDA8) surface ozone for air quality applications: a case study with GEOS-Chem v9.02. *Geosci Model Dev* **2019**, *12* (8), 3641-3648. DOI: 10.5194/gmd-12-3641-2019.

(6) Guo, J. J.; Fiore, A. M.; Murray, L. T.; Jaffe, D. A.; Schnell, J. L.; Moore, C. T.; Milly, G. P. Average versus high surface ozone levels over the continental USA: model bias, background influences, and interannual variability. *Atmos Chem Phys* **2018**, *18* (16), 12123-12140. DOI: 10.5194/acp-18-12123-2018.

(7) Lee, H. M.; Park, R. J. Factors determining the seasonal variation of ozone air quality in South Korea: Regional background versus domestic emission contributions. *Environ Pollut* **2022**, *308*. DOI: 10.1016/j.envpol.2022.119645.

(8) Marais, E. A.; Jacob, D. J.; Wecht, K.; Lerot, C.; Zhang, L.; Yu, K.; Kurosu, T. P.; Chance, K.; Sauvage, B. Anthropogenic emissions in Nigeria and implications for atmospheric ozone pollution: A view from space. *Atmos Environ* **2014**, *99*, 32-40. DOI: 10.1016/j.atmosenv.2014.09.055.

(9) Stevenson, D. S.; Dentener, F. J.; Schultz, M. G.; Ellingsen, K.; van Noije, T. P. C.; Wild, O.; Zeng, G.; Amann, M.; Atherton, C. S.; Bell, N.; et al. Multimodel ensemble simulations of present-day and near-future tropospheric ozone. *J Geophys Res-Atmos* **2006**, *111* (D8). DOI: 10.1029/2005jd006338.

(10) Philip, S.; Martin, R. V.; Snider, G.; Weagle, C. L.; van Donkelaar, A.; Brauer, M.; Henze, D. K.; Klimont, Z.; Venkataraman, C.; Guttikunda, S. K.; Zhang, Q. Anthropogenic fugitive, combustion and industrial dust is a significant, underrepresented fine particulate matter source in global atmospheric models. *Environ Res Lett* **2017**, *12* (4). DOI: 10.1088/1748-9326/aa65a4.
